# Gamification and Cognitive Factors: Research Hotspots, Knowledge Structure, and Future Directions Based on Bibliometric Analysis

**DOI:** 10.3390/jintelligence14070150

**Published:** 2026-07-17

**Authors:** Deao Song, Jien Guo, Xuaner Rao, Xinyu Hu, Xinyuan Gu, Junming Chen

**Affiliations:** Faculty of Humanities and Arts, Macau University of Science and Technology, Taipa, Macau 999078, China

**Keywords:** gamification, cognitive factors, bibliometric analysis, CiteSpace, digital learning, cognitive training, digital health, cognitive load

## Abstract

Gamification increasingly influences learning experiences, cognitive engagement, and behavioral performance in digital learning, cognitive training, and health intervention contexts. However, the mechanisms underlying cognitive factors, along with related research hotspots and evolutionary trends, have not been adequately synthesized. Using the Web of Science Core Collection, this study analyzes 813 publications on gamification and cognitive factors published between 2012 and 2024. Using a bibliometric method, CiteSpace was utilized to analyze publication trends, collaborations, keyword co-occurrence, cluster structures, burst terms, cited references, and knowledge-map visualizations. The cluster analysis produced 10 interrelated themes: “flipped classroom,” “active learning,” “continuance intention,” “dementia,” “executive function,” “cognitive control training,” “computational thinking,” “cognitive training,” “cognitive load” and “user experience”. Potential future directions suggested by the bibliometric patterns include: (1) expanding gamification across educational contexts; (2) refining gamification theory models that focus on cognitive processes by examining user experience and cognitive load as potential mechanisms that link gamification design features to outcomes such as motivation, self-efficacy, task performance, and continuance intention; (3) promoting applications in cognitive training, cognitive impairment intervention, and digital health; (4) optimizing experimental design, data collection, interdisciplinary collaboration, and personalized design; and (5) clarifying how gamification shapes cognitive processes such as attention allocation, cognitive load regulation, problem solving, executive function, and computational thinking. This study does not aim to establish causal effects; rather, it uses bibliometric evidence to reveal the developmental trajectory, thematic structure, and emerging directions of research on gamification and cognitive factors.

## 1. Introduction

### 1.1. Research Background and Motivation

Gamification is defined as the intentional application of game design elements into non-game settings to produce gameful experiences and enhance engagement, persistence, and task performance (Deterding et al., 2011a; Ortiz-Rojas et al., 2025). As digital education, human-computer interaction, and intelligent learning environments are being developed, the value and potential of gamification in promoting cognitive outcomes have attracted increasing scholarly attention. Gamification has been used extensively in research into cognitive factors. For example, in education and online learning, gamified design is widely applied to improve learners’ attention, cognitive engagement, and knowledge retention, which can induce sustained involvement in learning processes through task goals, immediate feedback, and reward mechanisms (Deterding et al., 2011b; Hamari et al., 2014; M. Li et al., 2023; Sailer & Homner, 2020). In cognitive training and assessment, gamification has been used to increase the enjoyment and sustained engagement of experimental and training tasks and therefore to facilitate the measurement and training of cognitive abilities, including attention, executive functions such as working memory and attentional control, and processing speed (Khaleghi et al., 2021; Lumsden et al., 2016). Gamification is frequently employed in health promotion and rehabilitation training to improve users’ adherence to intervention tasks and their willingness to engage in repeated practice, facilitating individuals to complete cognitive, behavioral, or rehabilitation training in a more interactive and goal-oriented environment (Johnson et al., 2016; Tosto-Mancuso et al., 2022).

Gamification is also used in organizational training and professional learning to simulate work-related tasks and training scenarios. Through goal setting, progress feedback, and contextualized challenges, gamification helps learners develop knowledge retention, problem-solving abilities, and decision-making skills. In human-computer interaction, mobile application research, and online platform research, gamification mechanisms have been applied to shape users’ information selection, attention allocation, and continuance behavior, thus affecting cognitive processes in digital settings (Bitrián et al., 2021; Koivisto & Hamari, 2019). These applications and studies extend the practical boundaries of gamification in education, training, health, and human-computer interaction and suggest that gamification is more than a motivational tool. Rather, it may influence cognitive processing and, in turn, learning and behavioral performance. Looking at future development, a market research report forecasts that the global gamification market is expected to grow from US$36.46 billion in 2026 to US$112.32 billion in 2031, at a compound annual growth rate of 25.24% for the forecast period (Mordor Intelligence, 2026). Given the rapidly increasing demand for applications and the ever-closer relationship between gamification and cognitive research, it is necessary to systematically review the trajectory, knowledge base, and developmental trends in the research on the effect of gamification on cognitive factors.

The research foundation for examining the effects of gamification on cognitive factors can be traced back to the early twenty-first century, when related studies primarily focused on digital game-based learning, instructional games, and the effects of video games on attention, learning motivation, and cognitive performance (Garris et al., 2002; Gee, 2004; Green & Bavelier, 2003). However, most of these early studies focused on complete or serious games rather than offering a clear conceptualization of “gamification” as a distinct design approach. Their emphasis was more often on learning, interest, and engagement, while explanations of the underlying cognitive processing mechanisms remained relatively limited.

The rapid development of the Internet, mobile communication technologies, and online learning platforms led to the inclusion of more explicit gamification design elements (e.g., points, badges, leaderboards, progress bars, immediate feedback, and task-based challenges) in educational and training settings (Deterding et al., 2011a, 2011b). These elements have, to some extent, enhanced learners’ learning engagement, behavioral participation, and academic performance, and have provided new possibilities for investigating the impact of gamification on cognitive engagement, knowledge retention, and learning performance (Landers, 2014; Sailer & Homner, 2020). At the same time, researchers have begun to examine the potential cognitive load of gamified design. For example, complicated reward systems, competitive mechanisms, or interface design may contribute to extraneous cognitive load, reducing learning outcomes (Plass et al., 2015; Turan et al., 2016).

Cognitive load theory is an important framework for understanding how gamification can affect cognitive processes. The theory assumes that human working memory capacity is limited and that both task complexity and information-processing demands that are irrelevant to learning goals affect learning and problem-solving performance (Paas et al., 2003; Sweller, 1988; Sweller et al., 1998). In gamified contexts, design elements like points, badges, leaderboards, instant feedback, and task challenges do not automatically enhance cognitive performance. When these elements serve to communicate task goals, organize key information, and provide feedback on task performance, they may help learners direct their limited cognitive capacity to processing relevant to the task. On the other hand, rules that are too complex, too many cues for competition, or redundant interface information can be sources of extraneous cognitive load and may hamper the selection and integration of the relevant information (Sweller, 2011; Sweller et al., 1998).

Working memory, inhibitory control, and cognitive flexibility are important prerequisites for maintaining attention, updating information, and switching between tasks from the perspective of executive functions (Diamond, 2012). Hence, the association between gamification design and learning or behavioral performance should not be attributed solely to increased motivation. Moreover, the effects of gamified design on the distribution of attention, working memory processing, executive control requirements, and cognitive load should be considered. A meta-analysis has indicated that gamification may be associated with cognitive, motivational, and behavioral learning outcomes; however, these outcomes are not equivalent and should not be interpreted through a single theoretical framework (Sailer & Homner, 2020).

Recent advances in artificial intelligence, learning analytics, and adaptive technologies have opened new research avenues in gamification, allowing systems to offer personalized feedback, dynamic challenges, and tailored reward pathways based on learners’ behavioral data, cognitive levels, and individual differences (Lumsden et al., 2016; Xiao & Hew, 2024). However, these developments increase the need for a theoretical basis for the design of gamification, data privacy protection, the digital literacy of learners, and the assessment of cognitive effects (Francis et al., 2023; Q. Liu & Khalil, 2023).

Critical challenges in digital learning, cognitive training, and sustained user engagement, such as maintaining attention, fostering cognitive involvement, and enhancing learning outcomes, can be addressed with gamification. Therefore, it can contribute to knowledge retention, problem solving, and continued learner involvement (Lumsden et al., 2016; Sailer & Homner, 2020). With the help of systematic and visualized bibliometric analysis, we can gain a comprehensive understanding of the application status, knowledge structure, research hotspots, and developmental trends in studies on the influence of gamification on cognitive factors, which can guide future theoretical development and practical application (Aria & Cuccurullo, 2017; C. Chen, 2006).

Therefore, this study explores the development of research on gamification and cognitive factors from different perspectives, including publication trend, country and institutional cooperation, co-occurrence of keywords, co-citation of documents, and research frontiers. The aim is to explore the potential of gamification to support cognitive engagement, manage cognitive load, support cognitive training, and optimize digital learning experiences. Therefore, this study provides both theoretical support and practical implications for the further application of gamified design in education, health, training, and cognitive measurement and assessment (López-Belmonte et al., 2020; Trinidad et al., 2021).

### 1.2. Conceptual Scope of Cognitive Factors

In this study, the term “cognitive factors” primarily refers to cognitive processes, cognitive abilities and higher-order cognitive skills involved in the acquisition, processing, retention, regulation, and application of information by users in gamified environments. More specifically, these factors encompass: cognitive engagement, cognitive load, attention, working memory, executive function, cognitive control, and computational thinking. Cognitive engagement refers to the strategic thinking, deep processing, and self-regulatory investment that learners bring to the task. Cognitive load is the demand on information processing generated by task complexity, information presentation formats, and other processing demands. Executive function includes core processes such as working memory, inhibitory control, and cognitive flexibility (Diamond, 2012; Fredricks et al., 2004; Sweller et al., 1998). In this study, computational thinking is considered a higher-order problem-solving skill that includes decomposition, abstraction, algorithm development, and system design (Wing, 2006).

Furthermore, this study encompasses research contexts such as cognitive assessment, cognitive training, interventions for cognitive impairment, dementia, and digital health. However, these contexts are not defined as “cognitive factors” themselves. Rather, they represent specific application areas where gamification is used to assess, train, or support cognitive processes and abilities. For example, research on dementia and other cognitive impairments reflects the application of gamification in cognitive screening, cognitive training, and health interventions, rather than treating disease status itself as a cognitive factor (Bahar-Fuchs et al., 2019). Conversely, motivation, self-efficacy, satisfaction, user experience, and continuance intention are not included within the core scope of ”cognitive factors” in this study. These constructs are included only when they co-occur with explicit cognitive processes, cognitive abilities, or cognition-related application outcomes. They are treated as experiential, motivational, or behavioral variables associated with the use of gamified systems. Specifically, user experience mainly concerns users’ subjective perceptions and evaluations of the interaction process and system characteristics (Hassenzahl & Tractinsky, 2006), whereas continuance intention primarily reflects users’ behavioral intention to continue using an information system after initial use (Bhattacherjee, 2001). This distinction explains why “user experience” and “continuance intention” appear in the cluster results: they reflect connections among related research topics and do not imply that they are conceptually equivalent to cognitive factors.

### 1.3. Research Gaps

The extensive body of literature on the influence of gamification on cognitive factors requires systematic analysis to clarify the current state of research, the knowledge structure, and potential future trends. Several review studies have examined the development of gamification research. Seaborn and Fels (2015) conducted a systematic review of theoretical and empirical studies on gamification, outlining its core concepts and directions for application in interactive systems and user experience research. Dicheva et al. (2015) summarized the application characteristics of gamification in education through a systematic mapping study, showing that points, badges, leaderboards, and feedback are among the most common design elements in educational gamification. Sailer and Homner (2020) further examined the effects of gamification on cognitive, motivational, and behavioral learning outcomes in a meta-analysis and found a significant positive effect on cognitive learning outcomes. Furthermore, Lumsden et al. (2016) systematically reviewed the application of gamification in cognitive assessment and cognitive training, demonstrating that gamified approaches have been used in tasks related to attention, working memory, executive function, and other cognitive abilities.

However, existing review studies have mainly focused on the educational effects, motivational mechanisms, theoretical foundations, or specific application contexts of gamification. There remains a lack of a systematic visual analysis of the overall knowledge structure, core research communities, thematic evolution, and frontier trends regarding the relationship between gamification and cognitive factors. As a statistical and quantitative method, bibliometric analysis is well-suited to processing large-scale literature data. It can reveal publication trends, knowledge structures, collaboration networks, and emerging topics in a given research field, thereby, to some extent, reducing the limitations of subjective selection and narrative synthesis in traditional reviews (Donthu et al., 2021). Although previous research has conducted bibliometric analyses of the broader field of gamification and revealed its research frontiers, knowledge structure, collaboration networks, and hotspot themes (Trinidad et al., 2021), bibliometric studies specifically focusing on the influence of gamification on cognitive factors remain relatively limited.

Bibliometric analysis uses bibliographic data, including publications, authors, keywords, citations, and references, to map research output, collaboration structures, thematic associations, and intellectual foundations using quantitative and network-based methods (Donthu et al., 2021; Zupic & Čater, 2015). Its findings are shaped by database coverage, search strategies, and the quality of bibliographic data, and therefore primarily reflect structural and relational patterns within the selected body of literature. As a methodological limitation, bibliometric analysis cannot directly assess the risk of bias in individual studies or infer causal relationships between gamification and cognitive outcomes (Hernán & Robins, 2020; Martín-Martín et al., 2018).

Bibliometric analysis, unlike systematic reviews, does not evaluate the quality of individual studies or synthesize findings within the context of a specific research question. Instead, it offers a field-level view of research development and knowledge structure. Conversely, systematic reviews use predefined procedures for searching, screening, and appraising studies to synthesize existing evidence (Page et al., 2021). Bibliometric analysis can analyze co-authorship, keyword co-occurrence, and co-citation relations in large bodies of literature using standardized bibliographic data and explicit computational rules. This allows for the identification of the development trajectory of a field, the major research communities, the conceptual structure and intellectual base (Cobo et al., 2011; Donthu et al., 2021; Zupic & Čater, 2015). While this does not remove researcher judgment in the selection of data or interpretation of results, clear documentation of search procedures, data processing, and parameter settings can reduce reliance on selective reading and narrative synthesis and increase the transparency and reproducibility of the analytical process (Donthu et al., 2021; Zupic & Čater, 2015).

Compared with meta-analysis, bibliometric analysis does not extract or statistically pool effect sizes from primary studies and therefore cannot estimate an overall effect or assess between-study heterogeneity. Conversely, meta-analysis quantitatively synthesizes effect sizes when study designs and outcome measures are sufficiently comparable (Nakagawa et al., 2023). Bibliometric analysis does not require diverse application contexts, gamification designs, and cognitive outcomes to be converted into directly comparable effect sizes. Therefore, it is suitable for examining literature sets that include heterogeneous research populations, intervention formats, task paradigms, and outcome measures (Donthu et al., 2021; Nakagawa et al., 2023; Zupic & Čater, 2015). Moreover, it can integrate collaboration relationships, thematic co-occurrence, intellectual bases, and temporal patterns to illustrate the connections and evolution of different research traditions within an interdisciplinary field (C. Chen, 2006; Cobo et al., 2011).

Because the purpose of this study is not to estimate the average effect of a specific gamification intervention, but to identify the collaboration network, thematic evolution, and intellectual base of research on gamification and cognitive factors, bibliometric analysis was adopted as a field-level analytical approach. The study aims to provide an overall structural overview of this research area and to offer guidance for subsequent systematic reviews and meta-analyses focused on specific gamification mechanisms or cognitive outcomes. Based on these considerations, CiteSpace was used to visualize the collaboration network, keyword co-occurrence, co-citation relationships, research hotspots, and emerging trends in this field (C. Chen, 2006).

CiteSpace is an important tool for visualizing scientific literature and analyzing knowledge maps. It can identify research hotspots, knowledge bases, and emerging trends through co-citation analysis, keyword co-occurrence, burst-term detection, and network-structure analysis (C. Chen, 2006). Therefore, this study employs bibliometric methods and CiteSpace software to conduct a comprehensive, quantitative, and visual analysis of the literature on the influence of gamification on cognitive factors. The findings are expected to provide useful references for practitioners and researchers to understand better the current status, knowledge base, and future directions of research on gamification and cognitive factors.

### 1.4. Research Objectives and Significance

This study systematically analyzes 813 publications from 2012 to 2024 to reveal progress and trends in research on the influence of gamification on cognitive factors, identify key contributors and research hotspots, and construct a knowledge framework for this field. Specifically, the objectives of this study are to identify influential authors, institutions, and countries; map the field’s intellectual structure; clarify major research themes; and propose future research directions and improvement strategies.

The significance of this study lies in its contribution to both the theoretical development and the practical application of gamification research on cognitive factors. By providing a systematic bibliometric overview, this study offers evidence to understand how gamification has been investigated in relation to cognitive engagement, cognitive load, cognitive training, and learning performance. Furthermore, the findings may help identify emerging commercial opportunities, inform policymaking, promote interdisciplinary research, and guide future technology- and application-oriented innovations. Overall, this study provides theoretical support for the development of gamified education and for gamified intervention or therapeutic practices and therefore has both academic value and social significance.

## 2. Research Methods

**Figure 1:**
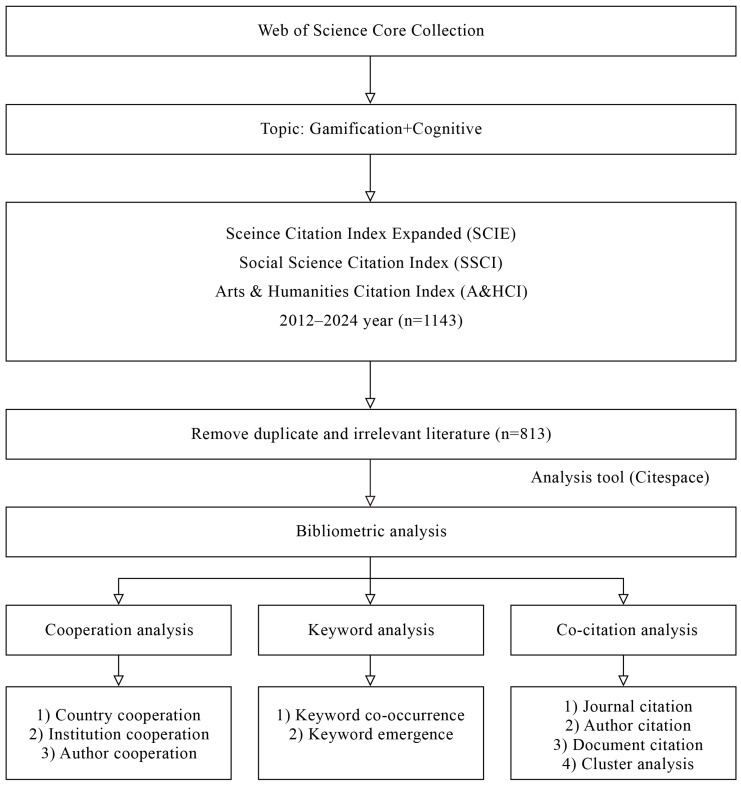
Research framework.

Bibliometrics is a methodological approach to quantitatively analyzing bibliographic data to reveal the structure, dynamics, and patterns of scientific research. Common bibliometric techniques are co-word analysis and document co-citation analysis (C. Chen, 2006). Bibliometric analysis is concerned with structured bibliographic information such as publications, authors, keywords, citations, and references. It can be used to trace the developmental trajectory, collaboration networks, thematic structure, and intellectual base of a research field through quantitative and network-based analyses (Broadus, 1987; Zupic & Čater, 2015). Its analytical validity is partly based on the fact that different bibliographic relationships reflect different structural dimensions of a field: co-authorship relations indicate research communities and collaboration structures, keyword co-occurrence reveals conceptual associations among topics, and co-citation relations help identify the intellectual base of a field and its evolution over time (Cobo et al., 2011; Zupic & Čater, 2015). Its reliability is mainly based on traceable data sources, explicit rules for search and data processing, and reproducible analytical procedures. When the data, parameter settings, and analytical steps are fully reported, the results can be analyzed and replicated by other researchers (Donthu et al., 2021; Öztürk et al., 2024). Thus, the present research triangulates the collaboration structure, thematic evolution, and intellectual base of research on gamification and cognitive factors using co-authorship, keyword co-occurrence, burst detection, and co-citation analyses. The purpose is to systematically clarify the research content of gamification and cognitive psychology, as well as their potential future directions for research. The general research framework is illustrated in Figure 1.

The collaboration analysis is conducted at the macro and micro levels, including country-, institutional-, and author-level collaborations. The keyword analysis primarily includes co-occurrence and burst-term analyses. The co-citation analysis includes cited-journal, cited-author, cited-reference, and document-clustering analyses.

### 2.1. Software Selection and Parameter Definition

The Web of Science database has generated more than 5000 records related to “gamification” over the last two decades, and many related studies have been published since the COVID-19 pandemic (Santos et al., 2024). Researchers have examined the influence of gamification on cognitive factors. The analysis and reading of such a large amount of literature to understand the historical development of research on gamification and cognition, as well as to identify research hotspots, are time-consuming and laborious. Furthermore, the researchers are inevitably limited by their own experience, memory, and reading ability, which may cause a relatively biased interpretation of the historical development and prospects of a scientific field. Therefore, bibliometric reviews can offer a more objective and comprehensive view of the historical developments, research hotspots, and developmental trends of a field by tracing changes in academic output, unlike traditional reviews that mainly rely on the viewpoints of scholars.

CiteSpace, developed by Chaomei Chen, is a Java-based software tool for data visualization and analysis. By processing and analyzing large-scale bibliometric data, it constructs co-citation-based network maps and detects emerging trends and transient patterns in the scientific literature (C. Chen, 2006). To date, many scholars have used CiteSpace to review the literature across various disciplines, including cognitive psychology. For example, W. Li et al. (2021) used CiteSpace to construct knowledge maps and analyze the literature on the effects of traditional Chinese health exercises on cognitive function, thereby revealing research hotspots and future directions in the field. J. Liu et al. (2020) conducted a CiteSpace-based visualization analysis to examine research on the cognitive processing of emotional words across different tasks. The integration of bibliometrics and CiteSpace provides researchers with a convenient and efficient tool to more accurately identify research hotspots and future directions for specific topics (G. Xu et al., 2024). Therefore, this study uses CiteSpace, version 5.7.R5, to conduct an in-depth analysis of the current state of research on gamification and cognitive psychology. Furthermore, Microsoft Excel 2022 was used for bibliometric analysis and visualization.

When constructing knowledge maps, “nodes” and “links” are two basic elements. The size of a node usually represents its influence or strength, whereas the thickness of a link indicates the closeness of the relationship between nodes. This study introduces the parameter “*k*” to highlight the main structure of the network. In the visualization process, the value of *k* controls node selection so that only countries, institutions, or authors with relatively high relevance or influence are displayed on the map. Thus, the study aims to more effectively identify and present key nodes and relationships within research collaboration networks.

Furthermore, two primary indicators are used in this study. Betweenness centrality measures the importance of a node in a network by indicating how often the node serves as an intermediary on the shortest paths between other nodes. When a node’s betweenness centrality exceeds 0.1, it is generally considered to play a key bridging role in the network. Burst strength measures changes in the frequency of a keyword or term over a specific period. A high burst strength indicates that the term has attracted rapidly increasing attention and may represent an emerging research hotspot or future research direction.

#### 2.1.1. Co-Occurrence Network

A co-occurrence network is a fundamental component of knowledge-map visualization. Scientific collaboration is defined as the simultaneous involvement of multiple authors, institutions, or countries/regions in a single publication (C. Chen, 2004). Because scientific research often requires extensive collaboration, examining patterns of scientific collaboration can reveal the research status and structural characteristics of a specific scientific field. Such collaboration can be analyzed along three dimensions: authors, institutions, and countries/regions.

When publications in a given research field are imported into CiteSpace as a dataset, collaborative relationships and scientific concepts can be represented as co-occurrence networks. CiteSpace uses color-coded nodes and links to distinguish merged networks across different time slices. The color of a network link indicates the year in which the co-occurrence relationship was first established. Nodes are composed of annual rings of different colors, and the thickness of each ring represents the frequency of co-occurrence in that year. A red ring indicates a citation burst in that year, reflecting a rapid increase in attention. A purple ring indicates the degree of betweenness centrality. Nodes with high betweenness centrality are meaningful because they often serve as bridges connecting different parts of the network.

#### 2.1.2. Burst Analysis

According to Kleinberg’s burst-detection theory, streams of documents, such as emails or academic articles, may exhibit a sudden spike in attention to a particular topic during a specific period, followed by a gradual decline. These temporal shifts in topics are identified using text-mining algorithms and represented as bursts of activity (Kleinberg, 2002). Based on Kleinberg’s algorithm, citation bursts are commonly used as indicators of active or emerging research topics (C. Chen, 2006).

A citation burst is a detected burst event that may last for several years or just one year. CiteSpace provides burst-detection functions for categories, keywords, and references (C. Chen et al., 2010). A citation burst indicates a sharp increase in the citation or occurrence frequency of a particular category, keyword, or reference. In other words, the corresponding topic, keyword, or publication has attracted strong attention from the scientific community during a specific period.

#### 2.1.3. Cluster Analysis

CiteSpace offers clustering functions based on titles, abstracts, and keywords, grouping publications into conceptual clusters with distinct research characteristics (C. Chen et al., 2014). Depending on the time-slicing settings, cluster maps can show how these clusters evolve. Furthermore, timeline views can clearly show the rise and fall of a given cluster over time and its connections with nodes in other clusters.

### 2.2. Data Collection and Processing

The Web of Science Core Collection (WoSCC) is a comprehensive database of academic literature that provides high-quality bibliographic data for scientific research. Scholars have extensively used it as a reference data source for bibliometric analysis. Therefore, WoSCC data were collected and processed by CiteSpace for further bibliometric analysis.

We performed a literature search in the Web of Science Core Collection (WoSCC) on 15 May 2025. The search strategy is presented in Table 1. The search was performed in the Science Citation Index Expanded (SCI-EXPANDED), the Social Sciences Citation Index (SSCI), and the Arts & Humanities Citation Index (A&HCI). Publication period was restricted to 2012–2024. Only English-language journal articles were included. The first search resulted in 1143 records. The research team checked for duplicate records after export by DOI, title, and author information. No duplicate records requiring removal were identified, and all records were moved to manual screening.

Inclusion and exclusion criteria were specified before screening to increase transparency and reproducibility of the screening process. Screening of personnel, stages, and procedures in case of disagreements was specified (Page et al., 2021). The titles and abstracts of all retrieved records were screened independently by two researchers. Full texts were obtained and evaluated when the topical relevance could not be ascertained from the title and abstract alone.

Records were included only when they met both of the following criteria:

1. The study explicitly addressed gamification, defined as the use of points, badges, leaderboards, progress feedback, task challenges, or other game design elements in non-game contexts.
2. The research objective, theoretical framework, methodology, or findings directly involved at least one cognitive factor, cognitive process, or cognition-related outcome as defined in this study.

Records were excluded when they met any of the following criteria:

1. The study focused only on video games, serious games, virtual reality, or other digital learning technologies without explicitly incorporating or examining gamification design elements.
2. Cognition-related terms were mentioned only in the background, keywords, or introduction and were not treated as a research focus, theoretical mechanism, measured variable, or study outcome.
3. The study primarily focused on emotional, motivational, market-related, physical health, or clinical symptom outcomes, without directly addressing the cognitive factors defined in this study.
4. The study adopted cognitive behavioral therapy as its primary clinical intervention framework and mainly assessed anxiety, depression, symptom relief, or treatment adherence without directly examining the relationship between gamification design and cognitive processes, cognitive abilities, or cognition-related outcomes.

For example, studies comparing the effects of video games, serious games, or virtual reality training that did not specify or analyze gamification design elements were excluded. Studies that mentioned the term “cognitive” only in the introduction but did not measure or discuss any cognition-related variables were also excluded. Similarly, clinical intervention studies focused on cognitive behavioral therapy that did not examine the relationship between gamification and cognitive factors were excluded.

When disagreements arose between the two reviewers, they first discussed the records against the predefined criteria. For records where consensus could not be reached, a third researcher independently reviewed them and made the final decision. Independent screening and predefined procedures for resolving disagreements can reduce potential bias associated with judgments made by a single reviewer (Waffenschmidt et al., 2019). Finally, 330 records that failed to meet one or more of the above criteria were excluded, leaving 813 publications for the subsequent bibliometric analysis.

### 2.3. Descriptive Statistical Analysis

#### 2.3.1. Publication Volume Analysis

**Figure 2:**
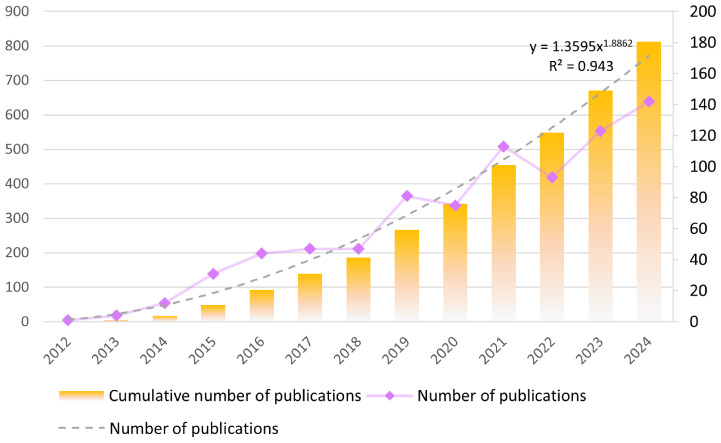
Cumulative annual publication volume and annual publication volume trend chart.

This study analyzed the annual publication trends for 813 publications on the impact of gamification on cognitive factors, as presented in Figure 2. The number of publications increased slowly but steadily from 2012 to 2018. During this period, researchers began to notice that incorporating computer-based and game-like components into students’ learning processes could effectively support learning under conditions of cognitive overload (Rai & Beck, 2012).

Between 2019 and 2024, the number of related studies and publications increased rapidly. One possible reason is that after the outbreak of the COVID-19 pandemic, many schools switched from face-to-face to online or remote learning. This shift required educators and institutions to adapt their teaching strategies quickly. This change has contributed to the rapid growth of research on gamified education and other digital learning approaches (Faudzi et al., 2022; Nieto-Escamez & Roldán-Tapia, 2021). The trend in publications continued upward in 2024.

#### 2.3.2. Category Analysis

**Figure 3:**
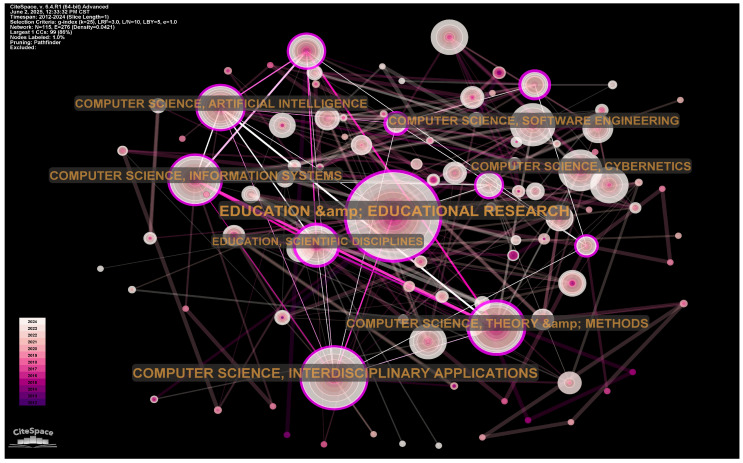
Visual analysis of research categories on improving cognitive factors through gamification.

The category analysis shows the disciplinary scope of a particular knowledge domain. The results based on the category analysis of the 813 publications are shown in Figure 3. The results suggest that Education & Educational Research and Computer Science are the most dominant disciplinary categories in this field and have important contributions to the overall knowledge structure.

The most cited article in the Education & Educational Research category is “Foundations of Game-Based Learning” by Plass et al. (2015). This study provides a theoretical foundation for game-based learning. It addresses how digital games can engage learners at affective, behavioral, cognitive, and sociocultural levels, providing an understanding of the cognitive implications of game-based and gamified learning environments.

The most-represented subcategory in the broad field of Computer Science is Computer Science, Interdisciplinary Applications, with 124 related articles. One of the most influential studies among these is “Engaging Asian students through game mechanics: Findings from two experimental studies” by Hew et al. (2016). Students who participated in the study were divided into two instructional groups, namely gamified and non-gamified, to examine the effects of gamified instructional approaches on cognitive learning factors. Furthermore, other subfields of computer science, namely Computer Science, Information Systems and Computer Science, Theory & Methods, have numerous publications and attracted considerable scholarly attention.

The disciplinary categories show that research on gamification and cognitive factors is predominantly located at the intersection of education and computer science. Other important research directions include domains such as Medical Informatics and Engineering, Electrical & Electronic. These disciplines have collectively contributed to research concerning the impact of gamification on cognitive factors.

#### 2.3.3. Journal Analysis

The analysis systematically evaluates journals based on publication output, citation frequency, impact factor, and research scope, thereby revealing their academic influence, research quality, and publication trends. As shown in Table 2, Lecture Notes in Computer Science published the most articles in this field, with 36 publications. It was followed by Frontiers in Psychology and Education and Information Technologies, with 18 and 17 publications, respectively. These results suggest that researchers in this field favor these publication venues and have made notable contributions to research on gamification and cognitive factors.

## 3. Collaboration Analysis

The analysis of collaboration can reveal the most active countries, institutions, and authors in a specific research field, as well as collaboration patterns among them (Gazni et al., 2012). Country-level collaboration analysis is employed in this section to identify the main countries contributing to research on the impact of gamification on cognitive factors at the macro-level. Then, the main research institutions at the meso level are analyzed through institutional collaboration analysis. Finally, at the micro level, author collaboration is analyzed to distinguish the major researchers and their collaboration relationships in this field.

### 3.1. Country Collaboration

**Figure 4:**
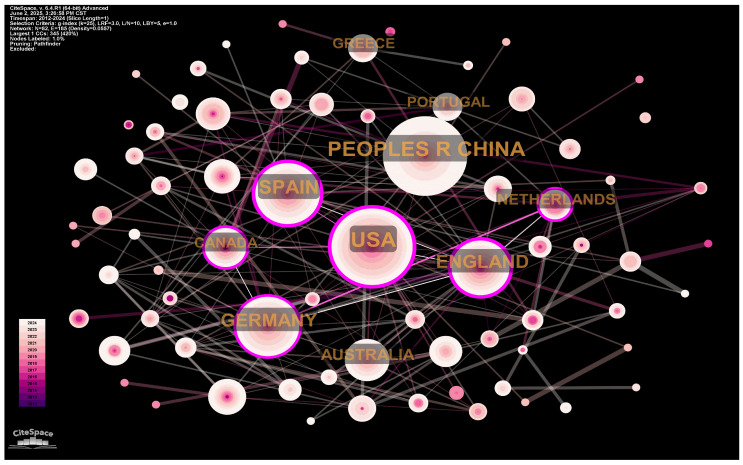
Country collaboration network.

Country collaboration analysis reveals publication distribution patterns across countries and offers a quantitative view of global academic exchange (Gazni et al., 2012). In this study, CiteSpace was used to conduct a country collaboration analysis, and Figure 4 presents the 10 countries with at least 25 publications. As shown in Figure 4, the collaboration network among countries or regions comprises 82 nodes and 185 links. The results indicate that multiple countries have formed relatively close collaborative relationships, suggesting the emergence of international research teams in this field.

In the network, the size of each node represents the number of publications from a given country. Conversely, the thickness of the links between nodes reflects the degree of collaboration between countries. When a node’s betweenness centrality exceeds 0.1, a purple ring is displayed around it, indicating the country’s importance and influence in the field. A larger purple ring indicates a stronger bridging role in the collaboration network. The statistical results show that 39 countries, including New Zealand, Australia, Canada, China, and India, have betweenness centrality values greater than 0.1, indicating their relatively important academic positions in this research area. Among them, the United States has the highest betweenness centrality of 0.47, indicating that it plays a central role in connecting researchers from multiple countries in the field of gamification and cognition.

A total of 85 countries contributed to this field. This study selected the top 10 countries by publication output and presents the results in Table 3. China had the most publications, with 138 articles, followed by the United States and Spain, with 133 and 79 articles, respectively. This country distribution is broadly consistent with findings from related bibliometric studies of gamification research. Studies on gamification in online education and computational thinking indicate that the United States, China, and Spain are among the leading contributors. In particular, Spain and China have demonstrated relatively high research output in studies on gamification and computational thinking (Triantafyllou & Sapounidis, 2026; Yazdi et al., 2024).

The results further show that the Netherlands had the highest average number of citations per article, followed by the United States and Germany. Furthermore, this study collected H-index values for the top 10 countries. The United States had the highest H-index, indicating the strongest academic influence in this field, so the research profiles of these countries need to be further studied.

The Netherlands had the highest average citation frequency, at 24.14 citations per article. Among the top 10 highly cited publications from the Netherlands, with the lowest citation count at 23, eight were related to medicine or health care. To provide descriptive context, this study further examined highly cited publications associated with the Netherlands. The most highly cited papers primarily addressed e-therapy and serious games, participative healthcare services, interventions for children with attention-deficit/hyperactivity disorder, and cognitive bias modification for adolescents with substance-use problems (Boendermaker et al., 2015; Bul et al., 2015; Fleming et al., 2016; Hammedi et al., 2017). These publications indicate that highly cited Netherlands-associated research in the present dataset is more frequently situated in healthcare, mental health, and cognitive-intervention contexts.

### 3.2. Institutional Collaboration

**Figure 5:**
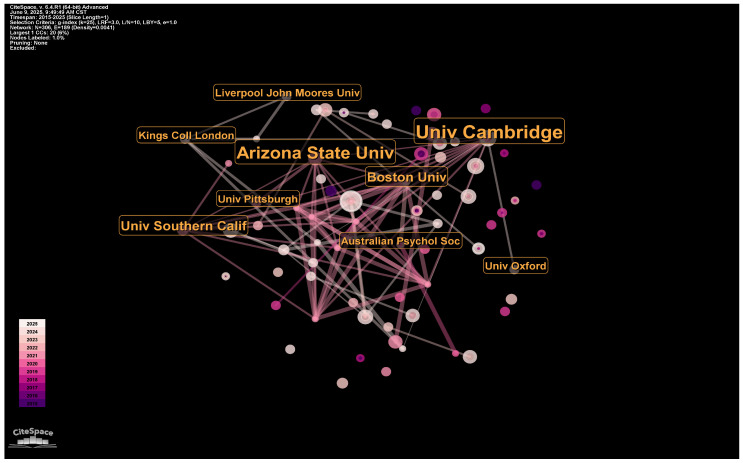
Institutional collaboration network.

Institutional collaboration analysis aims to deepen understanding of collaboration mechanisms, assess collaboration potential, optimize resource allocation, achieve mutual benefits, and promote the common development of participating institutions (Gazni et al., 2012). In this study, CiteSpace was used to analyze institutional collaboration, as shown in Figure 5. Figure 5 presents nine institutions with at least four publications. Most of these institutions exhibit relatively close collaborative relationships, which are conducive to the development of this field.

Several regional institutional collaboration clusters can be observed, including the Faculty of Education at the University of Hong Kong, the Faculty of Health and Social Sciences at the Hong Kong Polytechnic University, and the Hong Kong Polytechnic University. These clusters have promoted knowledge and resource sharing within regional academic networks. Furthermore, the participating institutions span multiple disciplines, including health care sciences and services (Kwan et al., 2024) and education (Ng et al., 2024). This indicates that research on the influence of gamification on cognitive factors is a multidisciplinary field that integrates education, health sciences, psychology, computer science, and related domains.

A total of 201 institutions contributed to this field. Table 4 lists the top 10 institutions by publication output. The most productive institution was the Faculty of Education at the University of Hong Kong, with 13 publications. Its primary research focus is gamified education and technology-enhanced learning (Hew et al., 2016; Ng et al., 2024; Xiao & Hew, 2024). Within this institution, many studies focused on the research team led by Khe Foon Hew, in collaboration with other scholars in the Mathematics, Science, and Technology academic unit.

The Monash University Faculty of Medicine, Nursing and Health Sciences and the Hong Kong Polytechnic University Faculty of Health and Social Sciences ranked second, each with five publications. At Monash University, the Addiction and Impulsivity Research Laboratory, led by Antonio Verdejo-Garcia, focuses on addiction, impulsivity, cognitive mechanisms, and intervention research. Related studies have examined cognitive bias modification and relapse prevention among individuals with alcohol use disorder (Manning et al., 2021; Verdejo-Garcia, 2023).

At the Hong Kong Polytechnic University Faculty of Health and Social Sciences, related research primarily focuses on gerontological nursing and cognitive health. Representative researchers, including Claudia K. Y. Lai and Rick Yiu Cho Kwan, have focused on dementia care, cognitive frailty, e-health, gerontechnology, and virtual-reality-supported or gamified motor-cognitive training for older adults (Kwan et al., 2024).

Meanwhile, King’s College London Institute of Psychiatry, National Taiwan Normal University College of Education, Penn Medicine, and the Perelman School of Medicine each published four articles and ranked fourth. The studies associated with these institutions span several areas, including gamified cognitive assessment, gamification-assisted experimental research, gamified education, cognitive load, and sustained participation in digital or VR-based learning environments (Juh et al., 2021; Malanchini et al., 2021). Furthermore, the University of Hong Kong Faculty of Education had the highest H-index. Conversely, the Tampere University Faculty of Information Technology and Communication Sciences had the highest average number of citations per article, indicating its leading influence in this field.

The most influential article from the University of Hong Kong Faculty of Education was “Investigating the Effects of Gamification-Enhanced Flipped Learning on Undergraduate Students’ Behavioral and Cognitive Engagement” (Huang et al., 2019). This study showed that incorporating gamified elements into flipped learning can enhance undergraduate students’ behavioral and cognitive engagement, providing evidence of the positive influence of gamification on students’ cognitive engagement.

### 3.3. Author Collaboration

**Figure 6:**
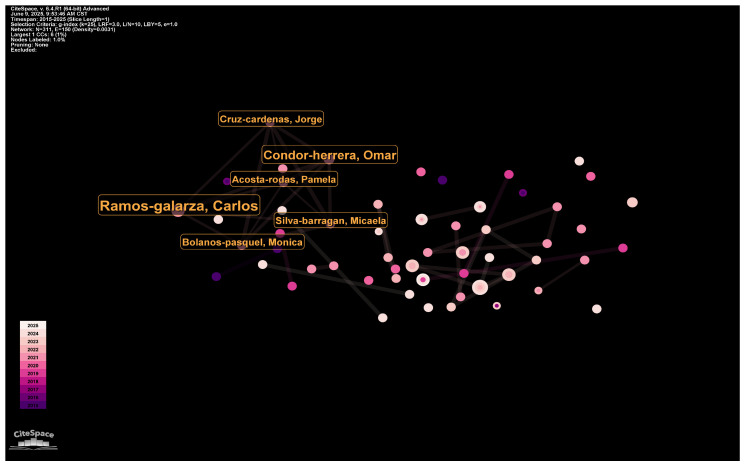
Author collaboration network.

Author collaboration analysis aims to reveal the structure of academic networks, collaboration patterns, and knowledge flows by examining co-authorship relationships among researchers (Gazni et al., 2012). In this study, CiteSpace was used to analyze authors with at least five publications, and the author collaboration network is shown in Figure 6. The results indicate that authors such as Ninaus M. and Hew K. F. have relatively high publication outputs in this field. Some authors have formed specific research groups, such as the team centered on Carlos Ramos-Romero.

The author cooperation network is generally characterized by low connectivity density and includes some relatively independent research groups and authors. This finding suggests that co-authorship ties among research teams remain limited in the literature included in this study.

Two possible reasons may be offered for the fragmented structure of the network. First, interdisciplinary and international collaborations usually have more organizational boundaries, communication processes, and geographical distances. Such conditions may increase the costs of coordinating projects and integrating resources, thereby leading teams to maintain existing collaboration relationships and reducing the likelihood of establishing new cross-team collaborations (Cummings & Kiesler, 2005).

Second, computer science and neuropsychology intersect in ways that often lead algorithm development to focus on performance indicators and behavioral data, while neuropsychology is more concerned with the validity and reliability of cognitive constructs, standardized assessment procedures, and clinical interpretability. Such differences may hinder direct comparison of task paradigms, data indicators, and standards for interpretation of findings, thereby adding to the difficulties of joint study design and research integration (Bauer et al., 2012). Furthermore, digital cognitive tasks used in different countries need to be adapted linguistically and culturally. This requirement can reduce the comparability of measurement results across samples and make standardization, comparison of results, and normative data development more difficult in multicenter studies (Nguyen et al., 2024). Furthermore, research on dementia, cognitive training, and executive function often contains sensitive health data. Data governance, privacy protection, and restrictions on cross-institutional data access may delay data sharing and external validation, limiting the feasibility of sustained international collaboration (Rieke et al., 2020).

Table 5 summarizes the publication records of 201 authors in this field and lists the top 10 authors by publication output. Among them, Ninaus M. ranked first, with eight publications. His most highly cited article was “Game Elements Improve Performance in a Working Memory Training Task” (Ninaus et al., 2015). This study examined whether game elements could enhance users’ performance and engagement in a working memory training task, thereby providing evidence for the potential cognitive benefits of gamified task design.

Other highly productive authors include Hew K. F. with seven publications; Chu S. K. W. and Hamari J. with six each; and Cheng Y. M., Chignell M., Qiao S., and Yeung S. S. with five each. Among them, Hew K. F. primarily focuses on gamified learning and technology-enhanced education (Lo & Hew, 2020). Conversely, Hamari J. has investigated users’ psychological and cognitive responses to gamification across various application contexts. Hamari J. had the highest average citations per article. His most highly cited article in this dataset was “Does Gamification Affect Brand Engagement and Equity? A Study in Online Brand Communities” (Xi & Hamari, 2020). Hamari J.’s research spans several directions, including gamification in marketing (Xi & Hamari, 2019b, 2020), gamified education, and the cognitive and psychological effects of gamification in virtual and digital environments. These directions are closely aligned with the field’s mainstream themes and therefore warrant further scholarly attention.

## 4. Keyword Analysis

Keyword analysis helps researchers understand research hotspots and changes in thematic structures within a discipline by revealing co-occurrence and burst relationships among terms (X. Chen et al., 2016). In this section, statistical and graphical methods are used to analyze keyword co-occurrence frequencies and keyword bursts in the literature to identify research trends and the knowledge structure of this field.

### 4.1. Keyword Co-Occurrence

Keyword co-occurrence analysis is primarily used to identify and analyze keywords that co-occur in academic literature, thereby revealing relationships among research topics and hotspots (Sedighi, 2016). In CiteSpace, the g-index served as the node-selection criterion, and the scaling factor *k* determined how many nodes to retain or exclude from the network. In this study, the g-index was used as the node-selection criterion, with the scaling factor set to k=25. Based on parameter-comparison tests, k=15 and k=25 retained more keywords with higher occurrence frequencies or stronger connections without making the network excessively dense. This setting therefore balanced information retention and map readability.

**Figure 7:**
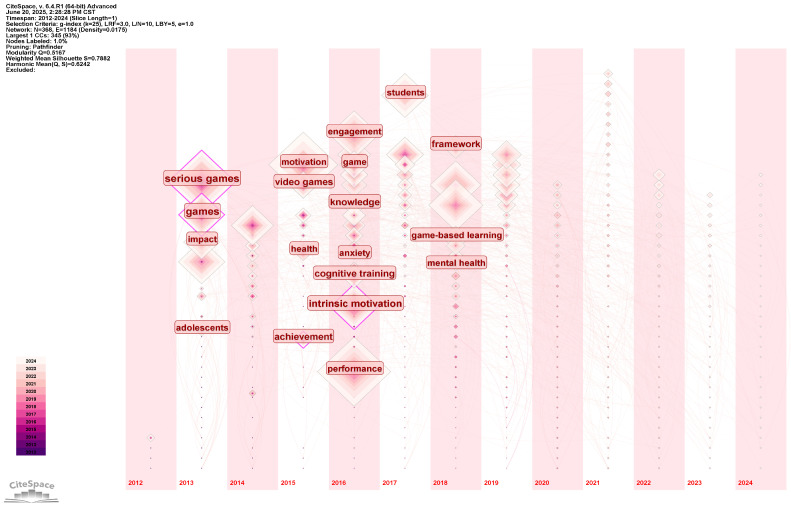
Keyword co-occurrence network time zone view.

A total of 364 keywords were identified from the 813 included publications. The keywords were then merged and cleaned to improve the consistency and accuracy of the analysis. Keyword co-occurrence analysis used one-year time slices to capture annual keyword occurrences, changes, and attention trends from 2012 to 2024. This approach enabled the identification of year-by-year thematic evolution within the research field. A time-zone view was generated, as shown in Figure 7.

As shown in Figure 7, several nodes are surrounded by purple rings, indicating that their betweenness centrality exceeds 0.1 and that they play important bridging roles in the network. Among them, the keywords “serious games” and “games” had the highest betweenness centrality values, reaching 0.18 and 0.17, respectively. “Intrinsic motivation” ranked third in betweenness centrality. Serious games are games that use entertainment to support objectives such as training, education, health, public policy, and strategic communication (Zyda, 2005). Intrinsic motivation is engaging in an activity for its inherent satisfaction rather than for separable outcomes; intrinsically motivated individuals act because of the enjoyment or challenge involved, rather than because of external incentives, pressures, or rewards (Ryan & Deci, 2000). Other central keywords include “achievement” and “video games.” In this context, “achievement” can be associated with intrinsic motivation to accomplish, which refers to the satisfaction derived from mastering a task or achieving a goal (Noels et al., 2003).

Moreover, Figure 7 illustrates the evolution of research hotspots in this field. Keywords such as “serious games” and “motivation” have remained major topics of discussion. Other themes have developed around these two central topics, suggesting that researchers should continue to pay attention to them. Important keywords extending from these core themes include “cognitive tasks,” “self-determination theory,” and “action tendency,” all of which represent major concerns in current research. Self-determination theory is a major approach to the study of human motivation. Research on self-determination theory began in the 1970s and was formally developed by Edward L. Deci and Richard M. Ryan (Adams et al., 2017). “Action tendency” refers to an individual’s readiness or urge to perform a particular behavior, especially as a component of emotional response (Huang et al., 2019).

### 4.2. Keyword Burst Analysis

**Figure 8:**
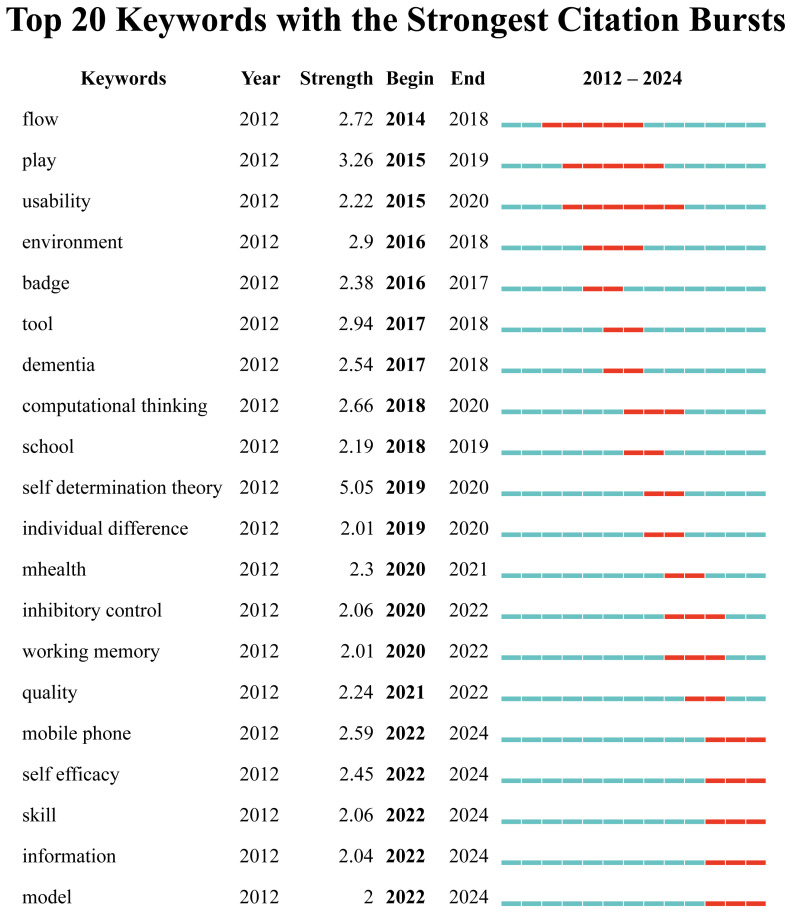
Top 20 keywords with the strongest citation bursts from 2012 to 2024.

Keyword burst analysis is a bibliometric method used to identify and trace changes in research hotspots and emerging trends within a specific field (X. Y. Liu et al., 2023). Figure 8 shows the burst strength of 20 keywords in the academic literature from 2012 to 2024. In the figure, the red line indicates the burst period of a keyword, during which its frequency increased significantly, suggesting that the topic received considerable scholarly attention. The dark blue line represents the overall time span from the beginning to the end of the analysis period, while the red segment marks the start and end years of the keyword burst. Based on the distribution of the red and blue lines, the burst periods of these keywords can be divided into three stages.

The first stage spanned 2014 to 2018, when research on the influence of gamification on cognition remained in an exploratory phase. The number of related studies was relatively limited, partly because gamification theory was still in its early stages of development (Rai & Beck, 2012). Meanwhile, users’ experiences with gamified systems had not yet been examined in depth. During this stage, the main burst keywords included “flow,” “usability,” “badge,” “environment,” “tool,” “dementia,” “school,” and “computational thinking.”

Among these keywords, “flow,” “usability,” and “badge” indicate that researchers at this stage paid considerable attention to the functions, implementation methods, and effects of gamification (Xi & Hamari, 2019a, 2020). As a core concept, “usability” received sustained attention from the early to the middle stages of this field. This suggests that researchers were concerned not only with the motivational appeal of gamified elements but also with how usability, ease of interaction, and user experience affect the completion of cognitive tasks (Savulich et al., 2017; Tong et al., 2016). Simultaneously, gamification elements, such as badges, leaderboards, points, and challenges, became important research topics in this field. As one of the key elements, “badge” attracted sustained scholarly attention because badges can transform task completion, staged progress, and competence achievement into visible rewards, thereby providing immediate feedback, enhancing users’ sense of achievement, and promoting continued participation through social recognition or status display mechanisms (Dicheva et al., 2015; Hakulinen et al., 2015).

Furthermore, studies on “dementia” indicate that the use of gamified and other non-pharmacological interventions in mental health and cognitive impairment treatment has gradually become a research hotspot (Savulich et al., 2017). However, research at this stage focused more on exploring the effects of gamification on health behavior change and digital interventions, rather than directly explaining cognitive mechanisms (Johnson et al., 2016; Rajani et al., 2019). Keywords such as “school” and “computational thinking” further suggest that gamification was considered to have positive effects on learning and motivation. Enhancing students’ engagement and attention through gamified design to improve educational outcomes became a relatively prominent topic (Imlig-Iten & Petko, 2018). Overall, during this stage, gamification was repeatedly discussed as an effective solution for improving learning and engagement. Nevertheless, how gamification changes cognition and which cognitive factors contribute to its effectiveness have not yet been fully examined (Hew et al., 2016).

The second stage spanned 2018 to 2021, marking an expansion in research on the influence of gamification on cognition. Building on the previous stage, a growing body of research further demonstrated the effectiveness of gamification in learning and instruction (Zainuddin et al., 2020). During this period, cognitive psychology and individual differences gradually attracted more scholarly attention. The main burst keywords in this period included “self-determination theory,” “individual difference,” “mHealth,” “inhibitory control,” and “working memory.”

Among these terms, “self-determination theory” is a widely studied theory of human motivation that encompasses both intrinsic and extrinsic motivation, as well as the relationships among motivation, growth, and well-being (Adams et al., 2017). “Individual difference” refers to diverse personal characteristics that significantly influence learners’ performance and attitudes in teaching and learning processes (Klock et al., 2020). The emergence of self-determination theory as a burst keyword in research on gamification and cognition suggests that scholarly attention has shifted from merely verifying the usability and effectiveness of gamification to examining how gamified design can more effectively guide or intervene in cognitive processes. The keyword “individual difference” further indicates that researchers increasingly recognize that users may respond differently to gamified elements, leading to a growing emphasis on specific populations, learner profiles, and personalized gamification.

The keywords “mHealth,” “inhibitory control,” and “working memory” together suggest that scholars began exploring the potential of gamification in addiction-related and health behavior interventions, particularly through its effects on cognitive functions such as inhibitory control and working memory (Forman et al., 2018). The research focus shifted from the earlier general concern with “dementia” toward more specific cognitive deficits and pathological mechanisms, such as impaired inhibitory control and working memory. Overall, the keyword changes in this stage indicate that research on gamification and cognitive factors gradually moved beyond early concerns with usability and broad health applications, turning instead toward specific cognitive functions, targeted user groups, and theory-driven intervention mechanisms.

The third stage occurred from 2021 to 2024, during which research on the influence of gamification on cognition entered a period of rapid development. During this period, gamification became increasingly popular worldwide. During this stage, “quality,” “mobile phone,” “self-efficacy,” “skill,” “information,” and “model” emerged as prominent research hotspots.

Among these keywords, “self-efficacy” is a relatively recent academic focus. Self-efficacy refers to individuals’ beliefs in their ability to organize and execute the actions required to achieve expected outcomes (Bandura, 2006). The emergence of this keyword suggests that scholars have begun applying gamification to long-term behavior change, habit formation, and goal-oriented interventions (Shadbad et al., 2023). The keyword “mobile phone” reflects growing attention to mobile-based gamified applications. Compared with relatively expensive and less convenient personal computers, smartphones are more affordable, widely accessible, and convenient, and thus may be more acceptable to participants in digital learning and intervention contexts (Faudzi et al., 2022).

Simultaneously, the keyword “quality” indicates that researchers are no longer concerned only with whether gamification can improve engagement but have begun to evaluate the quality of application content, interaction design, interventions, and empirical evidence. “Skill” suggests that gamification is increasingly used for skill development, clinical reasoning, professional competence, and learning improvement. “Information” indicates that research attention has gradually extended to information presentation, feedback mechanisms, and users’ processing of digital content. Finally, “model” reflects the growing emphasis on constructing theoretical models, mechanism models, and design frameworks in this field (Altomari et al., 2023; Mahmoudi et al., 2024). Overall, the keyword changes from 2021 to 2024 indicate that research on gamification and cognitive factors has shifted from early usability verification and engagement enhancement toward a stage characterized by mobile delivery, mechanism-oriented inquiry, model construction, and long-term intervention.

To summarize, the evolution of keywords in research on gamification and cognition clearly reflects research priorities and developmental trends at different stages. It also suggests the gradual emergence of two relatively distinct research trajectories: one focused on gamified education and the other on cognitive intervention and treatment. During the exploratory stage from 2014 to 2018, research hotspots centered on keywords such as “flow,” “usability,” “badge,” and “dementia.” At this stage, the two trajectories remained largely independent. Although gamification was still in its rapid phase of conceptual and practical development, studies from this period laid an important foundation for subsequent research. Subsequently, during the expansion stage from 2018 to 2021, research hotspots shifted toward “self-determination theory,” “working memory,” “individual difference,” and “inhibitory control.” This indicates that studies on the influence of gamification on cognition began to move toward individual differences and more specific cognitive domains. In the rapid development stage from 2021 to 2024, research attention further focused on keywords such as “quality,” “mobile phone,” “self-efficacy,” and “skill.” Overall, the diversity and complexity of research topics across these three stages demonstrate that research on gamification and cognition has gradually evolved from broad usability verification and engagement enhancement toward more refined, mechanism-oriented, and application-driven research directions.

## 5. Citation Analysis

Citation analysis is a method for evaluating the influence of academic literature. Counting citation frequencies for journals, authors, and publications can reveal research hotspots and developmental trends within a field (Cascajares et al., 2021). Among its techniques, document clustering analysis can identify similarities and differences among research themes. This section uses citation analysis to identify highly influential studies on the effects of gamification on cognition, thereby providing references for academic evaluation and future research directions.

### 5.1. Cited Conferences and Journals

**Figure 9:**
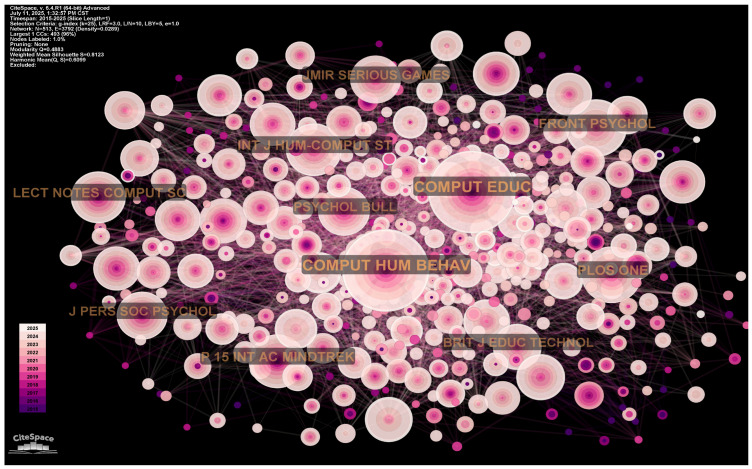
Cited journal and conference network.

Important conferences and journals play a key role in the development of this field. The nodes in Figure 9 represent journals or conference proceedings with at least eight publications, comprising a total of 10 sources. Figure 9 presents the cited journals and proceedings among the 813 related publications. It clearly and visually reveals the citation relationships within the research field, as well as the interdisciplinary integration and mutual influence among disciplines.

Figure 9 presents detailed information on the cited conferences and journals. The results show that articles in this field frequently cite studies published in journals such as Computers in Human Behavior, Computers & Education, and PLOS ONE. This indicates that these journals provide important disciplinary foundations and theoretical sources for understanding research on the influence of gamification on cognitive factors.

As shown in Table 6, Computers in Human Behavior ranked first, with 342 citations. According to the journal’s official aims and scope, Computers in Human Behavior examines computer use from a psychological perspective, focusing on both the use of computers in psychology-related fields and the psychological impact of computer use on individuals, groups, and society. Therefore, it provides a rich theoretical foundation for research on the cognitive effects of gamification. It was followed by Computers & Education and PLOS ONE, with 333 and 163 citations, respectively.

Among them, Computers & Education has published studies on the cognitive effects of gamified or game-based learning on students’ computational thinking (Y.-P. Cheng et al., 2023), as well as research on training systems, learning performance, and educational technology (Park et al., 2019). Conversely, PLOS ONE is a multidisciplinary journal that publishes studies on public health, cognitive assessment, and digital or mobile health interventions. For example, related studies have examined rapid online cognitive assessment in public health contexts (Baseler et al., 2022) and smartphone-based motivational interventions for brain health and lifestyle improvement (Roh et al., 2022).

### 5.2. Cited Author Analysis

**Figure 10:**
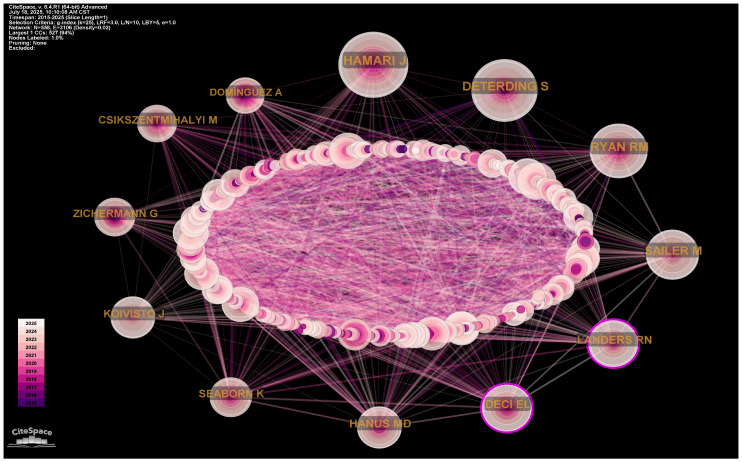
Co-cited author network.

Cited author analysis reveals the relationships and influence of different authors in academic research. It helps identify core scholars, potential collaboration opportunities, and interdisciplinary trends within a research field (Cascajares et al., 2021). In this study, CiteSpace was used to construct a co-cited author network. Figure 10 shows authors with at least 45 citations, including a total of 16 authors.

Among them, Hamari J. had the most prominent node and the highest citation frequency. He was followed by Deterding S. and Ryan R. M., who ranked second and third, respectively. These findings indicate that these authors have played important roles in shaping the theoretical foundations and research development of gamification and cognitive factors. The cited author network provides useful evidence for identifying influential scholars, potential collaboration opportunities, and interdisciplinary connections in this field.

As shown in Table 7, the most frequently cited author was Hamari J. His research spans multiple fields, including computer science, engineering, psychology, education and educational research, and business and economics. One of his most highly cited articles is “Does Gamification Work?—A Literature Review of Empirical Studies on Gamification” (Hamari et al., 2014). This article systematically reviewed empirical studies on gamification and provided an important foundation for understanding how gamification may influence users’ motivation, engagement, behavior, and cognitive responses. Therefore, it is regarded as a classic reference for research on gamification and cognitive factors.

The second most frequently cited author was Deterding S. His highly cited work, “The Lens of Intrinsic Skill Atoms: A Method for Gameful Design,” proposed a gameful design method based on intrinsic skill atoms (Deterding, 2015). Furthermore, Deterding et al.’s earlier work provided one of the most widely used definitions of gamification, namely the use of game design elements in non-game contexts (Deterding et al., 2011a). These studies have substantially contributed to the conceptual clarification and design foundations of gamification research.

Ryan R. M. ranked third among the cited authors. His research is closely related to psychology, education, educational research, and the social sciences. A representative highly cited article is “Self-Determination Theory and the Facilitation of Intrinsic Motivation, Social Development, and Well-Being,” co-authored with Deci (Ryan & Deci, 2000). This article explains the role of self-determination theory and intrinsic motivation in human development, education, health, and well-being, providing an important theoretical foundation for subsequent studies of motivation-related cognitive factors in gamified contexts.

Moreover, Landers R. N. exhibited the highest betweenness centrality among the cited authors, underscoring his bridging role in the co-citation network. His highly cited article, “An Inconvenient Truth: Arbitrary Distinctions Between Organizational, Mechanical Turk, and Other Convenience Samples,” addresses methodological issues in sampling and guides the design of more rigorous empirical studies (Landers & Behrend, 2015). Overall, the works and research directions of these authors are highly influential and warrant continued scholarly attention in the field of gamification and cognitive factors.

### 5.3. Cited Reference Analysis

**Figure 11:**
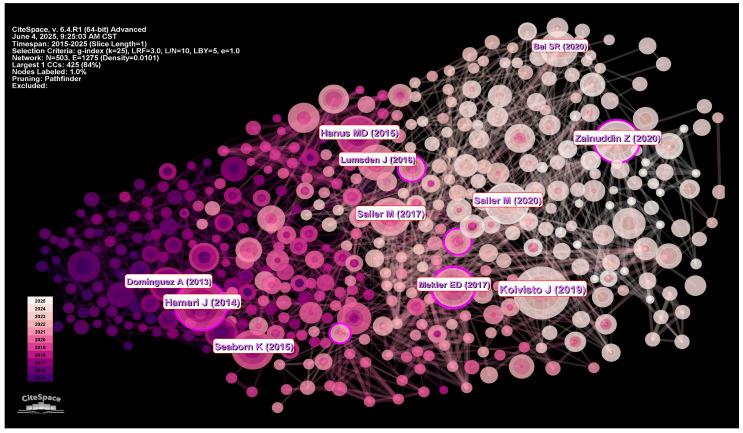
Cited reference network. The representative references include several highly co-cited studies in the network (Bai et al., 2020; Domínguez et al., 2013; Hamari et al., 2014; Hanus & Fox, 2015; Koivisto & Hamari, 2019; Lumsden et al., 2016; Mekler et al., 2017; Sailer et al., 2017; Sailer & Homner, 2020; Seaborn & Fels, 2015; Zainuddin et al., 2020).

Cited reference analysis not only reflects the academic influence, research quality, and scholarly recognition of publications but also serves as an important indicator for evaluating academic contributions (X. H. Liu et al., 2011). In this study, the cited references spanned 2015 to 2024. The cited reference network contained 503 nodes and 1275 links, as shown in Figure 11.

According to Figure 11 and Table 8, the most frequently co-cited article was “Foundations of Game-Based Learning,” published in Educational Psychologist by Plass, Homer, and Kinzer (Plass et al., 2015). The article examines the close relationship between game-based learning and cognitive engagement. It proposes a framework for understanding how games can engage learners at the affective, behavioral, cognitive, and sociocultural levels. It provides an important theoretical foundation for the systematic evaluation of cognitive factors in game-based and gamified learning environments, thereby improving the effectiveness and quality of gamified education.

The second-ranked article was “The Gamification of Learning: A Meta-analysis,” published in Educational Psychology Review by Sailer and Homner (Sailer & Homner, 2020). This study systematically synthesized empirical evidence on the effects of gamification on cognitive, motivational, and behavioral learning outcomes. It provided a methodological and theoretical foundation for subsequent research in this field.

The third-ranked article was “Does Gamification Affect Brand Engagement and Equity? A Study in Online Brand Communities,” published in the Journal of Business Research by Xi and Hamari (2020). This study examined whether gamification in online brand communities can enhance users’ brand engagement, loyalty, and identification, thereby providing a solid theoretical basis for its application in marketing contexts. Combined with the results in Table 5 and Table 7, Hamari J. appears in both the top 10 most productive and most cited authors, indicating that his study warrants continued attention from scholars in the fields of gamification and cognitive factors.

### 5.4. Cluster Analysis

**Figure 12:**
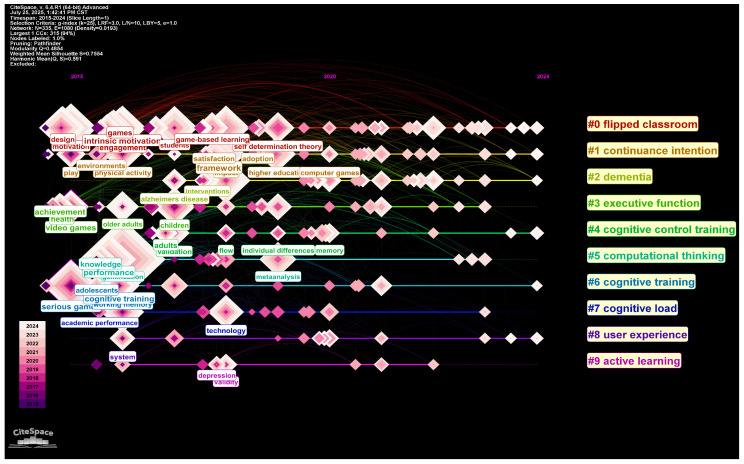
Cluster analysis of research on gamification and cognitive factors.

Cluster analysis can identify knowledge groups with strong thematic associations within a research field and reveal structural relationships and developmental patterns among research topics (C. Chen et al., 2014). As shown in Figure 12, keywords were extracted from the 813 included publications, and cluster analysis was conducted using two-year time slices. Compared with keyword co-occurrence and burst-detection analyses, cluster analysis focuses more on identifying relatively stable structural relationships among research themes. To determine an appropriate time-slice setting, this study compared clustering results obtained using one-year and two-year time slices. The one-year setting provided a more detailed representation of annual thematic changes, but some thematic clusters were relatively dispersed. Conversely, the two-year setting aggregated keyword co-occurrence information from adjacent years with related themes, resulting in more concentrated major clusters and clearer boundaries. Therefore, two-year time slices were used in the cluster analysis to improve the overall readability and interpretability of the results. Keyword co-occurrence and burst-detection analyses retained one-year time slices to present annual changes in research themes. A total of 10 thematic clusters were identified: Cluster #0, “flipped classroom”; Cluster #1, “continuance intention”; Cluster #2, “dementia”; Cluster #3, “executive function”; Cluster #4, “cognitive control training”; Cluster #5, “computational thinking”; Cluster #6, “cognitive training”; Cluster #7, “cognitive load”; Cluster #8, “user experience”; and Cluster #9, “active learning.”

To ensure consistency between the figure and the accompanying discussion, all 10 clusters are retained and discussed in this study. Some closely related themes are grouped within the same subsection because they share similar research objects or application contexts. For example, Cluster #0 (“flipped classroom”) is discussed together with Cluster #9 (“active learning”), whereas Clusters #3 (“executive function”), #4 (“cognitive control training”), and #6 (“cognitive training”) are grouped together. This organizational approach is intended to highlight thematic connections and does not indicate that the original clusters were deleted or merged. In particular, Cluster #7 (“cognitive load”) and Cluster #8 (“user experience”) are discussed separately as mechanism-related themes that link gamification design, cognitive processing, and behavioral outcomes.

Among these clusters, the “flipped classroom” cluster had the highest number of citations, indicating that it represents a major hotspot in this field. Since its emergence in 2015, the flipped classroom has gradually become a key component of research on the influence of gamification on cognitive factors. As the research on gamification has continued to develop and attract increasing scholarly attention, “continuance intention” has attracted broad interest among researchers. In recent years, as research in this field has deepened, the clusters of “dementia” and “cognitive control training” further indicate that studies on the influence of gamification on cognition have become increasingly refined and specialized. The following sections discuss each cluster in detail.

#### 5.4.1. Clusters #0 and #9: Flipped Classroom and Active Learning

In Cluster #9, “active learning” is closely tied to the theme of flipped classrooms. Flipped classrooms typically move content delivery to the pre-class phase and use class time for discussion, collaboration, case analysis, practical tasks, and problem-solving activities (Bishop & Verleger, 2013). A scoping review further identified a clear pedagogical connection between flipped and active learning: by reorganizing learning time inside and outside the classroom, flipped learning creates opportunities for learners to engage in interactive, application-oriented, and problem-solving activities (R. Li et al., 2023). In this context, gamification elements such as points, task goals, staged feedback, and visible learning progress may support learners’ participation in pre-class, in-class, and post-class learning activities. Findings reported by Lo and Hew on cognitive engagement and timely completion of learning tasks are consistent with this research focus (Lo & Hew, 2020). Therefore, in the present cluster map, “active learning” is interpreted primarily as a theme related to learner engagement and task-driven learning processes in gamified flipped classrooms.

Within this thematic context, existing studies have combined flipped classrooms with gamified instruction and examined their relationships with learning engagement, task involvement, and learning performance across disciplines, including computer science and language learning. For example, Huang et al. incorporated gamified ranking mechanisms into a flipped classroom for a computer-related course. Their results indicated that combining gamification with flipped learning improved students’ cognitive engagement, thereby enhancing learning efficiency (Huang et al., 2019). In language learning, combining gamification with flipped classroom strategies has shown promising effects (Zou, 2020). For instance, Girmen and Kaya introduced digital storytelling and game-based activities into a Turkish-language course using the flipped classroom model. Their study suggested that such designs can enrich classroom learning, enhance students’ participation and engagement, and promote the development of multiple skills (Girmen & Kaya, 2019). Beyond higher-education contexts, flipped learning has also produced positive outcomes in secondary-school classrooms. For example, studies in mathematics education have shown that flipped classroom approaches can effectively improve students’ engagement in class (Lo & Hew, 2021).

Furthermore, gamification has demonstrated broad applicability as an intervention strategy beyond the flipped classroom. For example, to promote environmental protection, Hsu and Chen designed a gamified application and found that it positively affected users’ behavioral intentions, encouraging environmentally friendly actions (Hsu & Chen, 2021). Similarly, in tourism, Mileva et al. suggested that location-based gamification applications could enhance user engagement, strengthen tourists’ destination awareness, and support destination brand co-creation (Mileva et al., 2021). Overall, the integration of gamification and flipped learning shows broad application prospects in education, and gamification, as a method, has been successfully applied in many fields beyond education.

However, whether gamification can consistently improve cognitive factors as tasks become more complex and usage duration increases remains inconclusive. First, repeated exposure to the same gamified design over a long period may diminish novelty, leading to boredom and weakening intrinsic motivation. A longitudinal study further indicated that after the novelty effect declines, gamified elements in flipped-classroom contexts may increase cognitive load and reduce learning efficiency (Rodrigues et al., 2022). Furthermore, many existing studies have focused primarily on using gamification to support the learning of basic knowledge. Conversely, relatively few studies have examined its effects in more complex knowledge domains. From the perspective of cognitive load theory, Sweller argued that higher task complexity, or element interactivity, increases intrinsic cognitive load. If unnecessary gamified stimuli are added to already complex tasks, they may increase extraneous cognitive load and undermine learning efficiency (Sweller, 2010).

#### 5.4.2. Cluster #1: Continuance Intention

Continuance intention generally refers to a user’s subjective intention to continue using an information system, digital platform, or learning tool after initial adoption. It is an important behavioral outcome in the Expectation-Confirmation Model (ECM). The model proposes that expectation confirmation, perceived usefulness, and satisfaction jointly influence subsequent use decisions (Bhattacherjee, 2001; Limayem & Cheung, 2008). In research on gamification and cognitive factors, continuance intention is not an independent cognitive ability. Rather, it can be understood as a behavioral outcome linked to gamification design, user experience, cognitive processing, and motivational states.

Existing studies have primarily explained this outcome through technology acceptance and motivational theories. Self-determination theory and its subtheory, cognitive evaluation theory, focus on whether gamification elements may support intrinsic motivation and subsequent participation by satisfying users’ needs for autonomy, competence, and relatedness (Suh, 2015; Xi & Hamari, 2020; Y. Xu et al., 2020). A field experiment by Olsson et al. indicated that a higher level of gamification was associated with stronger intrinsic motivation, greater satisfaction, and higher intentions to continue using the application (Olsson et al., 2016). These frameworks help explain users’ subjective evaluations and behavioral decisions. However, when analysis is limited to motivational or acceptance-based perspectives, it may be insufficient to explain how gamification design relates to attentional allocation, working-memory demands, and information-processing processes.

Cognitive load theory offers a complementary perspective on this issue. It suggests that task complexity, information presentation, and stimuli unrelated to task goals can influence how limited working memory resources are allocated (Sweller et al., 1998). Accordingly, design elements such as points, badges, leaderboards, progress feedback, and task challenges do not automatically increase continuance intention. Their effects may depend on alignment among design elements, task goals, information structure, and user capabilities (Sailer & Homner, 2020). Designs that align with task goals, clarify action objectives, and provide timely feedback may support sustained attention and task-relevant processing. Conversely, complex rules, redundant visual stimuli, or excessive competitive cues may increase extraneous cognitive load and diminish the user experience. Hwang et al. found that appropriately designed inquiry-based mobile learning activities were associated with lower cognitive load and improved learning achievement (Hwang et al., 2013).

Recent studies have started to link cognitive load to continuance-related variables. Ma found that higher cognitive load was associated with lower confirmation of expectations, perceived usefulness, and satisfaction among MOOC learners (Ma, 2025). Zhu and Yang used cognitive load theory and flow theory to explain virtual-community users’ continuance intention to seek information (Zhu & Yang, 2023). Cheng further suggested that gamification may be associated with MOOC users’ continuance intention through experiential factors such as cognitive presence (Y.-M. Cheng, 2023). Therefore, continuance intention may be conceptualized as a potential pathway linking gamification design, cognitive load, attentional engagement, confirmation, satisfaction, or flow to subsequent use behavior. Direct empirical examinations of this pathway remain limited, and the theoretical approaches used in related studies appear relatively dispersed (Krath et al., 2021). Future research may combine behavioral logs, task-performance measures, response-time data, or eye-tracking indicators to more directly examine how gamification design relates to cognitive processing and continuance behavior.

#### 5.4.3. Cluster #2: Dementia

Dementia is not a single disease but an acquired, progressive syndrome of cognitive decline caused by brain injury or disease. Among the different types of dementia, Alzheimer’s disease is the most common and major form (Arvanitakis et al., 2019). Furthermore, mild cognitive impairment (MCI), as an intermediate stage between normal aging and dementia, has become an important focus of both research and clinical practice because of its relatively high risk of progression to dementia (Petersen et al., 2014). Gamified or game-based digital tools have been explored for cognitive assessment, training, and supportive interventions related to mild cognitive impairment (MCI) and Alzheimer’s disease. Their feasibility, acceptability, and clinical effectiveness remain subjects of ongoing investigation. More broadly, gamification has demonstrated considerable potential in cognitive rehabilitation and behavioral intervention (Saragih et al., 2022). For example, Consales et al. developed an active video game, GiocAbile, for children with cerebral palsy. Their pilot study evaluated the system’s usability, acceptability, and user experience, showing that the game was well received by the target users and had potential value in rehabilitation contexts (Consales et al., 2024). Another study by Kwan et al. developed a virtual-reality-based intervention informed by the close relationship between physical and cognitive functions in older adults. The results showed that virtual reality motor-cognitive training could improve or maintain physical and cognitive functions among older adults with cognitive frailty (Kwan et al., 2024). In the field of addictive behavior intervention, Manning et al. examined cognitive bias modification among patients with alcohol use disorder and showed that such technology-supported cognitive intervention could assist relapse prevention without producing serious adverse effects (Manning et al., 2021). These studies reported acceptability, feasibility, or preliminary effects in contexts such as pediatric rehabilitation, exercise interventions for older adults, cognitive training, and addiction-related cognitive interventions. They suggest that gamified or game-based digital tools warrant further exploration across various health intervention settings.

However, the application of gamification in health care still faces multiple challenges. At the methodological level, Ferreira-Brito et al. noted that some studies on video games for age-related cognitive impairment and dementia have limitations, such as small sample sizes and inadequate control-group designs, which may affect the reliability of the findings and require more rigorous experimental verification (Ferreira-Brito et al., 2021). Regarding interdisciplinary collaboration, Rajani et al. found that many gamified smoking-cessation applications on the market contain design limitations and therefore emphasized the need for deeper involvement of clinical and health-care professionals in the development process (Rajani et al., 2019). Regarding usability and long-term effects, immersive VR interventions for older adults may be affected by sensory and motor limitations, interaction-learning costs, device burden, and fatigue. Interface and interaction complexity can directly reduce acceptability and training dosage, while long-term follow-up evidence remains insufficient (Baragash et al., 2022; Schaumburg et al., 2025). Moreover, for patients with addictive behaviors, the risk of relapse after intervention has not received sufficient attention, and continuous rehabilitation assessment mechanisms remain underdeveloped (Lenzi et al., 2025).

To summarize, although gamification shows considerable potential in health care and cognitive intervention contexts, current research still has notable limitations in experimental rigor, interdisciplinary collaboration, user-centered design, and long-term follow-up. Future studies should advance the scientific development and clinical translation of this field through methodological optimization, multi-stakeholder collaboration, and sustained longitudinal research.

#### 5.4.4. Clusters #3, #4, and #6: Executive Function, Cognitive Control Training, and Cognitive Training

Cluster #3 (‘’executive function”), Cluster #4 (‘’cognitive control training”), and Cluster #6 (‘’cognitive training”) are discussed together because of their close ties across research populations, assessment tasks, and application contexts. Executive function generally involves higher-order cognitive processes such as attentional control, behavioral inhibition, working-memory updating, and goal-directed decision-making (Diamond, 2012; Miller & Cohen, 2001). Cognitive control training typically involves repeated practice on tasks with high executive-control demands and focuses on processes such as attentional control, inhibitory control, working-memory updating, and task switching. In comparison, cognitive training is a broader intervention concept that generally refers to structured practice targeting specific abilities, including memory, attention, processing speed, and executive function (Diamond, 2012; Karbach & Verhaeghen, 2014; Simons et al., 2016). These three clusters remain distinct themes in the cluster map. Their joint discussion in this section reflects thematic connections within the existing literature and does not suggest that they are conceptually equivalent.

In some studies of executive function assessment, gamification elements have been incorporated into traditional cognitive tasks to increase participants’ acceptance of repeated testing and to explore assessment formats that more closely reflect everyday interactive contexts. For example, Song et al. used a mobile app with gamified features to assess cognitive control in children and adolescents and compared results to traditional neuropsychological tests. The study found no significant difference between the two approaches, and most participants preferred the gamified testing format (Song et al., 2020). Under the specific sample and task conditions of that study, these findings indicate that gamified assessment can be acceptable and practically feasible. However, they are not equivalent in terms of measurement validity across cognitive domains, age groups, or clinical contexts. Similarly, McWilliams et al. employed tablet-based games for repeated cognitive assessment in older adults and reported high adherence and positive user feedback (McWilliams et al., 2021). These results imply that gamified or game-based tasks may serve as an exploratory format for repeated cognitive screening, but their diagnostic accuracy, long-term stability, and clinical applicability require further validation.

Clusters #4 and #6 reflect scholarly attention to the application of gamified tasks in cognitive control training, occupational training, and organizational assessment. In team collaboration and personnel assessment settings, some studies have applied gamification mechanisms to training or assessment tasks and reported positive findings on participant reactions, team-related outcomes, or organizational attractiveness (C. Cheng & Chau, 2022; Georgiou & Nikolaou, 2020). However, these findings primarily indicate the potential value of gamified formats in task acceptability, engagement experience, or implementation approaches. They should not be interpreted directly as evidence that gamification improves specific cognitive abilities. Gamification elements, training objectives, outcome measures, and participant characteristics may vary across tasks. It is therefore necessary to distinguish between enhanced task engagement and changes in cognitive ability.

Existing evidence also suggests that the effects of gamified tasks might differ across cognitive domains and user groups. The context of a gamified or game-like task can sometimes reduce sustained task control and produce discrepancies between experimental data and questionnaire responses in healthy adults and adults with attention-deficit/hyperactivity disorder, as Delisle and Braun showed (Delisle & Braun, 2011). Lumsden et al. found no significant difference in data completeness between the gamified and non-gamified conditions in long-term web-based cognitive testing (Lumsden et al., 2017). Hence, this cluster does not present a clear effect of gamification on executive function or cognitive training. Rather, it is indicative of a continued body of research investigating the use of gamification design for cognitive assessment, cognitive training, and the administration of repeated tasks. Future research should use more rigorous control designs and longitudinal follow-up to clarify the task types, cognitive domains, and user groups in which specific gamification elements may support or constrain cognitive training and assessment processes.

#### 5.4.5. Cluster #5: Computational Thinking

Computational thinking (CT) applies core concepts from computer science to problem solving, system design, and the understanding of human behavior. It encompasses a set of thinking tools that reflect the breadth of computer science (Shute et al., 2017). In educational research, gamified computational thinking courses are often used as experimental contexts to examine the effects of gamification on learners’ cognitive factors, particularly motivational dimensions. Some studies suggest that gamified computational thinking instruction is associated with learners’ motivation, learning engagement, and continuance intention; however, its effects may vary across instructional contexts and design approaches.

For example, Cheng et al. developed a game-based learning environment to teach computational thinking. The results showed that this approach effectively stimulated students’ intrinsic and extrinsic motivation and further enhanced their willingness to engage in self-directed learning in the course (Y.-P. Cheng et al., 2023). Research has shown that game-based or gamified learning activities can improve interest in learning, deeper thinking, and other variables in the learning process that are closely related to the quality of cognitive processing (Imlig-Iten & Petko, 2018). Another cognitive factor that has been studied extensively is cognitive load. In the context of serious-game-based learning, for example, Babusiak et al. measured students’ cognitive load using electroencephalography (EEG) and found that higher cognitive load was significantly associated with lower levels of knowledge acquisition (Babusiak et al., 2021).

Although previous studies have shown that gamification and game-based learning can be used in computational thinking education and may positively affect learning motivation, learning engagement, and computational thinking performance, the specific mechanisms underlying these effects remain unclear. A related meta-analysis showed that gamification has a relatively clear effect on learning motivation in programming education; however, its effects on cognitive load and thinking skills were inconsistent among studies. This implies that gamification does not work through a single pathway (Zhan et al., 2022). In the meantime, computational thinking research has indicated that game-based learning platforms, student-generated questioning strategies, and varying levels of gamification have been employed to support computational thinking learning. However, the specific mechanisms require further examination (Y.-P. Cheng et al., 2023; del Olmo-Muñoz et al., 2023). Therefore, future research should further clarify the relationships among the elements of gamification, learning motivation, cognitive load, and computational thinking performance, and use behavioral data, learning-process data, and physiological indicators to establish a clearer mechanistic model for gamified computational thinking education.

#### 5.4.6. Cluster #7: Cognitive Load

In Cluster #7, “cognitive load” indicates that related studies increasingly examine how task demands, information presentation, and interaction mechanisms introduced by gamification design may relate to users’ information-processing demands. Cognitive load theory holds that individuals have limited working-memory resources for information processing during learning and problem solving. Task complexity, the manner in which information is presented, and additional demands unrelated to task goals may all influence cognitive processing (Paas et al., 2003; Sweller et al., 1998). Accordingly, rather than treating points, badges, leaderboards, or feedback mechanisms simply as design elements that promote engagement, the research represented by this cluster emphasizes that the effects of gamification elements may depend on their fit with learning tasks, user capabilities, and platform functions. Wang and Kartika Sari found that when game mechanisms were a better fit for learning tasks, learners reported lower cognitive load and more positive learning outcomes (Wang & Kartika Sari, 2024). This result suggests that cognitive load may offer an important perspective on differences in the effects of gamification, but it does not imply that all gamification mechanisms reduce cognitive load.

Existing studies suggest that the relationship between cognitive load and gamified experiences is highly context-dependent. Shaban et al. investigated user experience, cognitive load, and training performance concurrently in a gamified working-memory training application for children with learning disabilities. In that study, under the specific application and small-sample conditions, lower-cognitive-load activities were associated with more positive user experiences and better training performance (Shaban et al., 2021). However, these findings may also be affected by users’ age, ability characteristics, task format, and application design. Therefore, they are not sufficient to demonstrate that gamification design produces stable and consistent cognitive effects across different populations and tasks.

Cognitive load should not be considered a single outcome of gamification from a design perspective. Instead, it can be seen as a possible mechanism that links design features, user experience, and task performance. Alexiou and Schippers proposed that game elements, user experience, motivation, and learning outcomes are related at multiple levels, and that these relationships may depend on user characteristics (Alexiou & Schippers, 2018). In gamified learning or training, clear task rules, appropriate challenge, task-related feedback, and concise interface information may help users understand task procedures and keep their attention on the core information. Rather, complicated rules, redundant stimuli, or mismatched competitive mechanisms are likely to increase information-processing demands and impair users’ positive evaluation of the interaction process. Such relations should be further experimentally studied on specific design elements.

Overall, Cluster #7 suggests that future research should move beyond the simple question of whether gamification affects cognitive load and instead identify the mechanisms through which such effects may occur. Cognitive load and user experience may be viewed as two important links between gamification design and cognitive or behavioral outcomes, reflecting users’ information-processing demands and subjective evaluations of the interaction process, respectively. Future research could integrate cognitive load theory, task–technology fit theory, and theories related to motivation or continuance behavior to examine how gamification design relates to learning engagement, task performance, and continuance outcomes through user experience and cognitive load.

#### 5.4.7. Cluster #8: User Experience

In Cluster #8, “user experience” indicates that related research has increasingly examined not only whether gamification design is associated with outcomes such as learning, training, or continuance behavior, but also how users subjectively experience and evaluate the interaction process. User experience is generally subjective, dynamic, and context-dependent. It encompasses users’ overall perceptions of system usability, sense of control, feedback quality, level of challenge, satisfaction, and emotional experience (Hassenzahl & Tractinsky, 2006; Law et al., 2009). Therefore, in gamified environments, the value of points, badges, leaderboards, task challenges, and feedback mechanisms does not depend solely on their inclusion in a system. Rather, it depends on whether users can understand, accept, and integrate these design elements into the specific task process. Existing research further suggests that different game elements may elicit distinct psychological and behavioral responses. Therefore, gamification should not be treated as a uniform design solution that automatically improves user experience (Mekler et al., 2017; Sailer et al., 2017).

Notably, the results of the cluster analysis indicate that user experience has been an important topic in research on gamification and cognitive factors. They do not demonstrate that a particular gamification design will inevitably lead to positive experiences or subsequent behavioral changes. Associations with user experience, satisfaction, or continued participation have been reported in studies across different application contexts. For example, Olsson et al. found that the level of gamification in a location-based application was related to users’ intrinsic motivation, satisfaction, and continuance intention (Olsson et al., 2016). Consales et al. assessed the usability, acceptability and user experience of an active video game system for children with cerebral palsy in a health and rehabilitation setting (Consales et al., 2024). Shaban et al. evaluated user experience, cognitive load, and training performance simultaneously in a gamified working-memory training application (Shaban et al., 2021). These studies together suggest that user experience can be an important dimension for evaluating the feasibility and use process of gamified applications. However, relevant findings may still vary according to task goals, user characteristics, interaction modes, and duration of use (Shaban et al., 2021).

Conceptually, user experience and cognitive load are related, but they are not the same thing. Cognitive load is primarily the cognitive resources users require to understand rules, process information, and perform tasks. Conversely, user experience is the user’s subjective evaluation of the interaction process, including perceived usability of the system, sense of control, quality of feedback, enjoyment, and satisfaction (Hassenzahl & Tractinsky, 2006; Law et al., 2009). Therefore, a lower cognitive load does not necessarily indicate a more positive user experience, and a highly challenging or immersive gamified design does not necessarily entail a lower cognitive load. Alexiou and Schippers stated that the components of game design, user experience, motivation, and learning outcomes can be connected at different levels and differ according to user characteristics and specific contexts (Alexiou & Schippers, 2018). Therefore, user experience can be regarded as a relevant process variable for understanding how users accept, interpret, and evaluate gamification design, considering cognitive load alongside when analyzing gamified applications.

Overall, Cluster #8 suggests that future research should examine how different gamification design features relate to learning engagement, task performance, or continuance behavior, with user experience and cognitive load as mediators. Subsequent studies could integrate user-experience frameworks, cognitive load theory, and theories of motivation or technology acceptance to clarify the roles of different constructs in the pathway from “design features–user experience and cognitive load–behavioral or cognition-related outcomes.” Experimental designs, longitudinal tracking, and multi-source data may be used to examine the applicability of this pathway across different user groups and application contexts.

## 6. Future Research Directions

**Figure 13:**
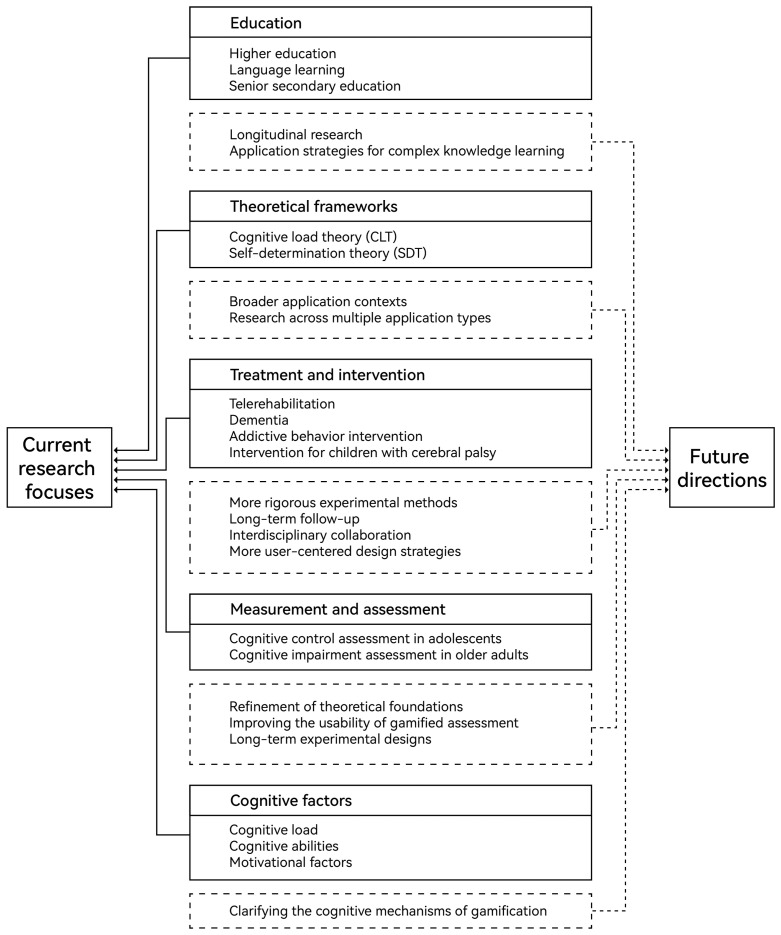
Key themes and future research directions.

Based on the keyword and citation analyses of the literature reviewed above, this study summarizes several key themes and future research directions, as shown in Figure 13. The following sections discuss these directions in relation to the bibliometric findings.

1. Expanding the boundaries of gamified education research.
  Research on learning motivation in gamified environments, as well as on more personalized and diversified learning content, has continued to increase. Clusters related to flipped classrooms, active learning, computational thinking, and cognitive load suggest that future studies may examine how gamification operates across different teaching domains, task difficulty levels, and learner groups. Particular attention should be paid to reducing the cognitive load associated with gamified design.
  Research across different subject domains, task difficulty levels, and learner groups can inform the development of more personalized and responsive educational approaches. At the same time, potential limitations should be considered. For example, long-term exposure to gamified systems may reduce novelty and interest, whereas complex gamified designs may increase extraneous cognitive load in more demanding learning contexts.
2. Refining theoretical models of gamification.
  Current research has drawn on cognitive load theory, self-determination theory, the Expectation-Confirmation Model, and self-efficacy theory to explain motivation, technology acceptance, continuance behavior, and cognition-related outcomes in gamified contexts. The keyword burst results, including “self-determination theory,” “working memory,” “inhibitory control,” “self-efficacy,” and “model,” together with clusters related to continuance intention, cognitive load, and user experience, suggest that future research may further integrate cognitive, motivational, and behavioral perspectives.
  In this direction, future studies may examine how different gamification elements are associated with attentional engagement, working-memory demands, inhibitory control, problem-solving performance, user experience, and continuance intention, while considering factors such as task complexity, rule clarity, feedback design, and interface information presentation. Cognitive load and user experience are important themes in the literature on gamification design, task processes, and cognitive or behavioral outcomes. Further research may more carefully examine how these theoretical perspectives complement one another in explaining the relationship between gamification design and cognition-related processes.
3. Applying gamification to the treatment and intervention of cognitive impairment.
  Clusters related to dementia, cognitive training, and cognitive control training indicate that gamification in cognitive training, cognitive impairment intervention, and digital health deserve continued attention. Future studies may further expand sample sizes and collect data through multiple channels, such as questionnaires, semi-structured interviews, behavioral measures, and physiological measurements. Longitudinal research may provide more reliable evidence on long-term effects.
  Addressing cognitive impairment through gamification is a complex multidisciplinary issue involving medicine, psychology, neuroscience, computer science, and design. Therefore, future research may benefit from stronger interdisciplinary collaboration. For individuals with cognitive impairment and older adults, the acceptability of gamification as an intervention method should be carefully considered. Interface design may need to align with the cognitive characteristics and preferences of different populations, for example, by simplifying game interfaces or using information visualization strategies.
4. Improving gamified cognitive measurement and assessment systems.
  The clusters of “executive function,” “cognitive control training,” “cognitive training,” and “dementia” indicate that gamified cognitive measurement and assessment deserve further attention. Future research may broaden its application in assessing adolescents’ cognitive control and evaluating cognitive impairment among older adults. Compared with traditional scales or single experimental tasks, gamified assessment can enhance participants’ engagement through task scenarios, immediate feedback, and interactive mechanisms, while collecting data such as reaction time, error rate, task completion, and sustained participation in a more natural interactive process.
  However, the scientific validity of gamified assessment tools still requires careful examination. Future studies may further evaluate their usability, accuracy, stability, and applicability across different time points, task difficulties, and user groups, thereby improving the reliability and practical value of gamified cognitive assessment.
5. Clarifying the mechanisms through which gamification influences cognitive factors in education.
  The cluster related to “computational thinking,” along with keywords such as “cognitive load,” “skill,” and “model,” indicates that the relationship between gamification and cognitive factors in education remains an important research direction. Existing studies suggest that gamification and game-based learning are associated with learning motivation, learning engagement, and computational thinking performance, but the specific pathways involved still require further examination. Because computational thinking involves complex processes such as decomposition, abstraction, algorithmic thinking, debugging, and problem solving, gamified elements may be examined not only as tools for increasing classroom interest but also as design features related to information processing, cognitive load, and problem-solving strategies.
  Future research may further distinguish the effects of specific gamification elements, such as points, badges, leaderboards, levels, and feedback mechanisms. Researchers may also examine whether gamification increases extraneous cognitive load in complex programming or algorithm-learning contexts, thereby affecting students’ understanding of core knowledge. Integrating behavioral data, learning-process data, questionnaires, interviews, and physiological indicators may provide a more detailed basis for understanding gamified computational thinking education.

## 7. Conclusions

This study analyzed 813 publications on gamification and cognitive factors in the Web of Science Core Collection from 2012 to 2024 using bibliometric analysis. The study used CiteSpace to analyze collaboration networks, keyword co-occurrence, burst detection, co-citation relationships, and thematic clusters. This study aimed to provide a field-level overview of the developmental trajectory, knowledge structure, major research communities, and thematic evolution of this research domain.

The results show that research on gamification and cognitive factors is highly interdisciplinary, spanning Education, Computer Science, Psychology, Health, and Rehabilitation. The collaboration networks show that the field has benefited from contributions by researchers across countries, institutions, and research teams. However, cross-institutional and interdisciplinary linkages among research teams remain relatively limited. The evolution of keywords also suggests a shift away from early field emphases on usability, badges, flow, and engagement experiences toward more context-specific and cognition-related topics, such as working memory, inhibitory control, individual differences, self-determination theory, mobile applications, and skill development.

Cluster and co-citation analysis further identified a set of related themes, including ‘flipped classroom’, ‘active learning’, ‘continuance intention’, ‘dementia’, ‘executive function’, ‘cognitive control training’, ‘cognitive training’, ‘computational thinking’, ‘cognitive load’, and ‘user experience’. The literature is distributed across two broad application contexts. The first is education and digital learning, including flipped classrooms, active learning, computational thinking, and related issues of learning engagement and task performance. The second is cognitive assessment, training, interventions for cognitive impairment, and applications in health and rehabilitation. Cognitive load and user experience emerged as important themes in the literature on gamification design, task processes, and cognition- or behavior-related outcomes. Continuance intention refers to academic interest in using digital platforms, satisfaction with them, and further participation.

The primary contribution of this study is to present a comprehensive overview of the development of research on gamification and cognitive factors across collaboration networks, thematic structures, intellectual bases, and research frontiers. The results are useful for understanding the core topics, interdisciplinary links, and the evolution of knowledge in this field.

## Figures and Tables

**Table 1:** Search strategy used in the Web of Science Core Collection.

Item	Details
Database	Web of Science Core Collection (WoSCC)
Citation Indexes	Science Citation Index Expanded (SCI-EXPANDED); Social Sciences Citation Index (SSCI); Arts & Humanities Citation Index (A&HCI)
Search Date	May 15, 2025
Search Field	Topic (TS)
Full Search String	TS=((gamification OR gamified OR gamifying OR gamif*) AND (cognitive OR “cognitive factor” OR “cognitive load” OR “cognitive function” OR “cognitive functions” OR “function, cognitive” OR “functions, cognitive”))
Timespan	2012–2024
Document Type	Article
Language	English

**Table 2:** Top 10 journals by number of publications.

Rank	Journals	Number
1	Lecture Notes in Computer Science	36
2	Frontiers in Psychology	18
3	Education and Information Technologies	17
4	JMIR Serious Games	16
5	Sustainability	14
6	Proceedings of the European Conference on Games-Based Learning	13
7	Edulearn Proceedings	12
8	INTED Proceedings	11
9	Computers in Human Behavior	10
10	Advances in Intelligent Systems and Computing	9

**Table 3:** Top 10 countries by publication output.

Rank	Country	Numbers	Citations	Average Citations	H-Index
1	China	138	1899	13.90	22
2	USA	133	2676	21.56	25
3	Spain	79	628	8.11	12
4	Germany	74	1258	18.24	15
5	England	66	888	13.45	19
6	Australia	40	484	12.10	13
7	Netherlands	28	676	24.14	13
8	Greece	27	268	9.93	9
8	Portugal	27	132	4.89	6
10	Canada	25	334	13.36	12

**Table 4:** Top 10 institutions by publication output.

Rank	Organization	Number	Citations	Average Citations	H-Index
1	The University of Hong Kong Faculty of Education	13	646	49.69	9
2	Monash University Faculty of Medicine, Nursing and Health Sciences	5	37	7.40	4
3	The Hong Kong Polytechnic University Faculty of Health and Social Sciences	5	20	4.00	2
4	King’s College London Institute of Psychiatry	4	56	14.00	2
5	National Taiwan Normal University College of Education	4	84	21.25	3
6	Pennsylvania Medicine	4	21	5.25	3
7	Perelman School of Medicine	4	21	5.25	3
8	Tampere University Faculty of Information Technology and Communication Sciences	4	283	70.75	2
9	The Education University of Hong Kong Department of Psychology	4	80	20.00	4
10	The Education University of Hong Kong Faculty of Education and Human Development	4	80	20.00	4

**Table 5:** Top 10 authors by publication output.

Rank	Author	Number	Citations	Average Citations	H-Index
1	Ninaus M	8	171	21.38	6
2	Hew KF	7	156	21.38	6
3	Chu SKW	6	95	15.83	5
4	Hamari J	6	338	56.33	5
5	Cheng YM	5	72	14.40	5
6	Chignell M	5	76	15.20	4
7	Qiao S	5	82	16.40	4
8	Yeung SS	5	82	16.40	4
9	Ahmad F	4	17	4.25	3
10	Ahmed Z	4	17	4.25	3

**Table 6:** Top 10 cited journals and proceedings.

Rank	Cited Journals	Citations
1	Computers in Human Behavior	342
2	Computers & Education	333
3	PLOS ONE	163
4	Frontiers in Psychology	162
5	International Journal of Human–Computer Studies	151
6	MindTrek ’11: Proceedings of the 15th International Academic MindTrek Conference: Envisioning Future Media Environments	144
7	Lecture Notes in Computer Science	140
8	Psychological Bulletin	123
9	British Journal of Educational Technology	122
10	JMIR Serious Games	118

**Table 7:** Top 10 cited authors by citation frequency.

Rank	Cited Author	Citations	Centrality
1	Hamari J	166	0.05
2	Deterding S	144	0.06
3	Ryan RM	116	0.10
4	Sailer M	92	0.05
5	Landers RN	83	0.15
6	Deci EL	81	0.13
7	Hanus MD	72	0.08
8	Seaborn K	65	0.05
9	Koivisto J	64	0.05
10	Zichermann G	56	0.06

**Table 8:** Top 10 cited references.

Rank	Reference	Citations	Author and Year
1	Foundations of Game-Based Learning (Plass et al., 2015)	669	Plass et al. (2015)
2	The Gamification of Learning: A Meta-analysis (Sailer & Homner, 2020)	506	Sailer and Homner (2020)
3	Does gamification affect brand engagement and equity? A study in online brand communities (Xi & Hamari, 2020)	225	Xi and Hamari (2020)
4	Investigating the effects of gamification-enhanced flipped learning on undergraduate students’ behavioral and cognitive engagement (Huang et al., 2019)	209	Huang et al. (2019)
5	Engaging Asian students through game mechanics: Findings from two experimental studies (Hew et al., 2016)	168	Hew et al. (2016)
6	Gamification of Technology-Mediated Training: Not All Competitions Are the Same (Santhanam et al., 2016)	158	Santhanam et al. (2016)
7	Maximizing the impact of e-therapy and serious gaming: time for a paradigm shift (Fleming et al., 2016)	131	Fleming et al. (2016)
8	Education for sustainable development through business simulation games: An exploratory study of sustainability (Gatti et al., 2019)	129	Gatti et al. (2019)
9	A comparison of flipped learning with gamification, traditional learning, and online independent study: the effects on students’ mathematics achievement and cognitive engagement (Lo & Hew, 2020)	128	Lo and Hew (2020)
10	Mental Health on the Go: Effects of a Gamified Attention-Bias Modification Mobile Application in Trait-Anxious Adults (Dennis & O’Toole, 2014)	126	Dennis and O’Toole (2014)

## Data Availability

Dataset available on request from the authors.
